# Targeted metabolomics to assess positive effects of empagliflozin in a Parkinson’s disease model: focused on the kynurenine pathway and oxidative stress

**DOI:** 10.1038/s41598-026-55891-1

**Published:** 2026-06-04

**Authors:** István Lénárt, Orsolya Horváth, Kinga Molnár, Csaba Bereczki, Péter Monostori, Péter Klivényi, Zsolt Galla

**Affiliations:** 1 Metabolic and Newborn Screening Laboratory, Department of Pediatrics, Albert Szent-Györgyi Medical School, University of Szeged, Szeged, 6725 Hungary; 2 Department of Neurology, Albert Szent-Györgyi Medical School, University of Szeged, Semmelweis u. 6, Szeged, 6725 Hungary; 3 Doctoral School of Clinical Medicine, University of Szeged, Korányi fasor 6, Szeged, 6720 Hungary; 4 HUN-REN-SZTE Neuroscience Research Group, Hungarian Research Network, University of Szeged Danube Neuroscience Research Laboratory, Tisza Lajos krt. 113, Szeged, 6725 Hungary

**Keywords:** Biochemistry, Diseases, Drug discovery, Neurology, Neuroscience

## Abstract

Sodium-glucose cotransporter 2 inhibitors, such as empagliflozin (EMPA), have been increasingly investigated for their potential neuroprotective properties, but their overall metabolic impact in Parkinson’s disease (PD) remains incompletely understood. Using a 1-methyl-4-phenyl-1,2,3,6-tetrahydropyridine (MPTP)-induced mouse model of PD, we investigated the effect of EMPA on tryptophan (TRP) metabolism, neurotransmitter levels and antioxidant markers in the striatum. Targeted ultra-high performance liquid chromatography tandem mass spectrometry (UHPLC–MS/MS) was used for metabolite quantification. Pairwise group differences were assessed using Welch’s two-sample *t*-test, with false discovery rate correction, and multivariate analyses were applied for exploratory pattern recognition. EMPA treatment significantly enhanced the neuroprotective arm of the kynurenine pathway (KP), increasing kynurenic acid (KA), anthranilic acid (AA), xanthurenic acid (XA) and the corresponding enzymatic activity ratios in MPTP-induced PD animals. The selective elevation of the KA/TRP ratio without a corresponding change in KYN/TRP suggests that EMPA acts specifically on the KAT-mediated neuroprotective branch, potentially through restoration of astrocytic redox state in the striatum, rather than through generalized modulation of IDO/TDO-driven TRP catabolism. In Sirtuin3 knock-out (S3KO) mice, EMPA reduced 3-hydroxykynurenine (3OHK) levels and the Oxidative Stress Index (3OHK/(KA + AA + XA)), and improved glutathione redox status, as reflected by reduced GSSG levels and an improved GSH/GSSG ratio. These results demonstrate that EMPA exerts significant neurometabolic effects in a mouse model of PD, shifting KP flux towards neuroprotective metabolites and improving redox homeostasis—particularly in the context of mitochondrial dysfunction modelled by Sirtuin3 deficiency. Future studies extending these findings to additional experimental models and clinical settings will be essential to fully elucidate the translational potential of EMPA in neurodegeneration.

**Supplementary Information:**

The online version contains supplementary material available at 10.1038/s41598-026-55891-1.

## Introduction

Parkinson’s disease (PD) is the second most common neurodegenerative disorder, affecting over 10 million individuals worldwide and imposing a substantial burden on healthcare^1^. Clinically, PD is characterized by the progressive degeneration of dopaminergic neurons in the substantia nigra (SN) and the accumulation of misfolded α-synuclein that aggregate into Lewy-bodies^2,3^. Although various mechanisms have been proposed regarding the etiology of PD, including genetic susceptibility, mitochondrial dysfunction, oxidative stress, and environmental exposures, its exact pathogenesis remains unclear^4^.

Numerous studies have reported altered tryptophan (TRP) metabolism in PD patients, both centrally and peripherally, playing significant role in the development and the progression of the disease^5–7^. TRP is an essential amino acid and a precursor of several bioactive molecules, involved in neurotransmission, cellular energy metabolism, and immune regulation^8^. More than 90% of TRP is metabolized through the kynurenine (KYN) pathway (KP), while smaller proportions are converted via the serotonin route^9^. Approximately 5% of dietary TRP is metabolized through the indole pathway by gut microbiota. The metabolites of this pathway affect the gut-brain axis and may play a role in the pathogenesis of neurodegenerative disorders^10^.

There is growing evidence that KP dysregulation is involved in the pathogenesis of several neurodegenerative diseases, including Alzheimer’s disease, Huntington’s disease and PD^11–13^. The KP produces several neuroactive metabolites with opposing effects: kynurenic acid (KA) exerts neuroprotective actions through antagonism at *N*-methyl-*D*-aspartate (NMDA) and α7-nicotinic acetylcholine receptors, whereas 3-hydroxykynurenine (3-OHK) and quinolinic acid (QA) are neurotoxic since they enhance oxidative stress and excitotoxic mechanisms^14–16^. Dysregulation of key KP enzymes such as indoleamine 2,3-dioxygenase (IDO), kynurenine monooxygenase (KMO), and kynurenine aminotransferases (KATs) can shift the metabolic balance toward neurotoxic metabolite production. In line, reduced KA levels and KAT activity have been reported in several brain regions of PD patients, supporting a pathological bias toward excitotoxicity and oxidative damage^17–19^. Evidence obtained from in vitro and in vivo models, as well as from affected patients, supports the involvement of the KP in PD pathophysiology and highlights its potential for identifying novel therapeutic targets.

Animal models are critical for elucidating the pathophysiology of PD and evaluating novel therapeutic strategies. Among neurotoxins, 1-methyl-4-phenyl-1,2,3,6-tetrahydropyridine (MPTP) is the most widely used in in vivo models due to its reliability to reproduce key pathological features of PD^20^. MPTP effectively crosses the blood–brain barrier (BBB) and is metabolized by monoamine oxidase-B (MAO-B) in astrocytes to form 1-methyl-4-phenylpyridinium (MPP⁺), which is selectively taken up by dopaminergic neurons through dopamine transporter (DAT). MPP⁺ inhibits mitochondrial complex I, leading to oxidative stress, energy failure, and neuronal death^21^. The MPTP model, beside its neurotoxic effect on dopaminergic neurons is also suitable for investigating metabolic master regulator families such as peroxisome proliferator-activated receptor-γ coactivator (PGC), and the silent information regulator 2 homologues (Sirtuin)-family^22–24^. Sirtuins are NAD⁺-dependent deacetylases that act as key metabolic and stress-response regulators across multiple cellular pathways. In particular, mitochondrial sirtuins such as Sirtuin3 (SIRT3) maintain energy homeostasis and mitochondrial function such as protection against oxidative stress through antioxidant enzyme superoxide dismutase 2 (SOD2) activation.

Empagliflozin (EMPA) is a selective sodium-glucose co-transporter 2 (SGLT2) inhibitor, with pleiotropic effects. It regulates glycemic control and also exerts anti-inflammatory, antioxidant, and mitochondrial-protective effects^25,26^. Emerging evidence indicates that EMPA may have neuroprotective effects in neurodegenerative disease models^27–29^.

**Fig. 1:**
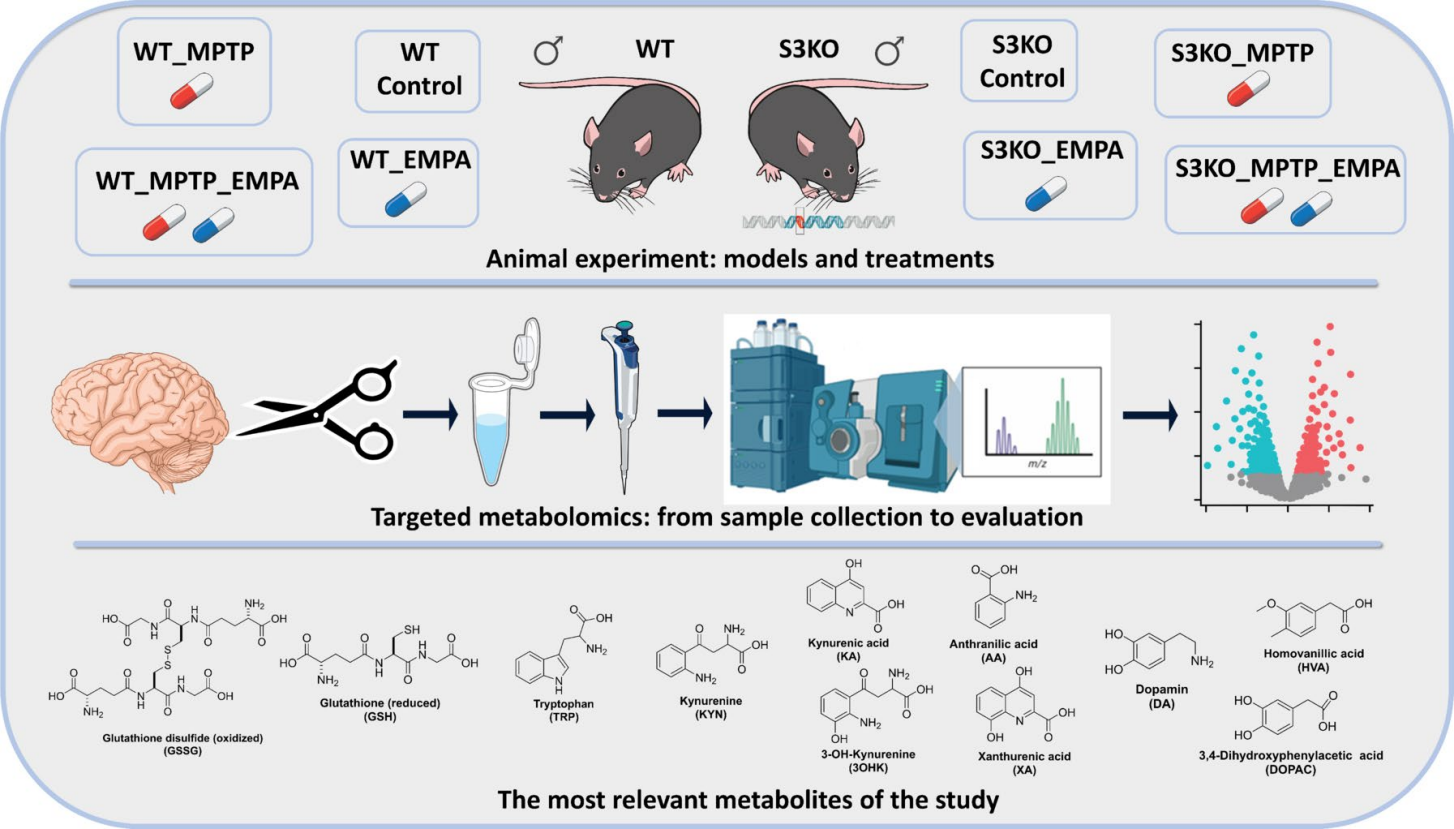
Schematic flow chart of the study. WT and transgenic S3KO mice were used for the animal experiments. Eight groups were created according to the MPTP and EMPA treatments. After sample collection and preparation, targeted UHPLC-MS/MS analysis and statistical evaluation were performed. The chemical structures represent the most relevant metabolites in this study. (WT: wild-type, S3KO: Sirtuin3 knock-out).

The aim of this study was to assess how MPTP treatment affects the TRP metabolism, neurotransmitters and glutathione antioxidant system. We also set out to investigate whether EMPA treatment is able to modify the alterations in these metabolic pathways. Targeted ultra-high performance liquid chromatography–tandem mass spectrometry (UHPLC-MS/MS) was used to quantify changes in neurologically relevant metabolite levels in the brains of control and MPTP-treated wild-type (WT) and SIRT3 knock-out (S3KO) animals (Fig. 1).

## Materials and methods

### Animal experiment

12-week old male Wild-type C57BI/6J (WT) and Sirtuin3 knockout (S3KO) (B6.129S6(Cg)-Sirt3^tm1.1Fwa^/J) mice were used in this study (21 ± 3 g). The animal strain was originally obtained from Jackson Labs (Jackson Laboratories, Bar Harbor, ME, USA).

Animals were randomly assigned to eight experimental groups: WT control *n* = 10, WT MPTP-treated *n* = 8, WT EMPA-treated *n* = 8, WT MPTP + EMPA-treated *n* = 9, S3KO control *n* = 5, S3KO MPTP-treated *n* = 8, S3KO EMPA-treated *n* = 6, S3KO MPTP + EMPA-treated *n* = 8. The widely used MPTP toxin-induced animal model was applied to induce PD-like disease. Animals received intraperitoneal injections of MPTP at a dose of 15 mg/kg, administered five times at 2-h intervals within a one day. The day after the final MPTP injection, animals were treated orally with 15 mg/kg EMPA once daily for 21 consecutive days. Striatal tissue was collected 22 days after the last MPTP injection and 24 h after the final EMPA administration.

Animals were randomly assigned to treatment groups by a blinded investigator, and no experimental confounders were identified. All mice were housed under standard conditions (12-h light/dark cycle, 23 ± 2 °C) with ad libitum access to regular chow and water.

Group sizes of *n* = 7–10 per group were targeted for WT background animals, consistent with sample sizes reported in comparable published MPTP mouse model metabolomics studies. For S3KO background groups, available animal numbers were constrained by the reproductive capacity of the S3KO colony, resulting in smaller group sizes (*n* = 5–9). All pairwise statistical comparisons were performed using Welch’s two-sample *t*-test, which does not assume equal variances or equal group sizes and is therefore appropriate for the unequal group sizes observed in this study. To further address the potential impact of small and unequal group sizes on the reliability of the reported findings, Cohen’s d effect sizes with bootstrap 95% confidence intervals were calculated for all primary endpoints, as described in the ‘effect size estimation’ section.

### Sample collection

Survival and body weight, general appearance, activity and signs of distress of the experimental animals were monitored daily. Survival rate was 97–98%, with maximum weight loss of 5%, no infections, wounding, internal bleeding, or chronic pain were observed. Mice reaching or exceeding the established euthanasia threshold were humanely euthanized by 5% isoflurane.

Otherwise, after 24 h of the last EMPA treatment, the animals were deeply anesthetized with inhalation of 2% isoflurane (Forane; Abott Laboratories Hungary Ltd., Budapest, Hungary). The depth of anesthesia was confirmed by assessment of reflexes. The thoracic cavity was surgically opened, a cannula was inserted into the left cardiac ventricle with concomitant incision of the right atrium, and the animals were subjected to transcardial perfusion with artificial cerebrospinal fluid (composition in mM: 122 NaCl, 3 KCl, 1 Na_2_SO_4_, 1.25 KH_2_PO_4_, 10 D-glucose, 1 MgCl*6H_2_O, 2 CaCl_2_*2H_2_O, 6 NaHCO_3_). The brains were rapidly excised on ice from the skull and stored at − 80 °C until analysis by UHPLC–MS/MS.

### Targeted quantification of metabolites by ultra-high performance liquid chromatography tandem mass spectrometry analysis

#### Sample preparation

Striatum tissue samples were prepared according to slight modifications of previously published and validated protocols^30–32^. Briefly, each striatal tissue specimen was weighed and homogenized in 3 × (v/w) volumes of ice-cold extraction solvent (97% LC–MS-grade H_2_O, 3% actonitrile 0.2% formic acid 0.02% ascorbic acid + internal standards) e.g., 90 µL water per 30 mg tissue, using an ultrasonic homogenizer (UP100H, Hielscher Ultrasound Technology, Germany; amplitude: 100%, cycle: 0.5). Protein precipitation was performed by adding acetonitrile + 0.2% formic acid + 0.02% ascorbic acid at a 1:19.2 ratio, followed by vortex mixing and centrifugation at 14,000 × g for 10 min at 4 °C. The resulting supernatant was collected, evaporated to dryness under nitrogen flow, and reconstituted in mobile phase prior to injection. All sample preparation steps were performed on ice to minimize metabolite degradation.

#### Reagents, reference standards, and internal standards

All reagents were of analytical or LC–MS grade. Acetonitrile (ACN) was obtained from Molar Chemicals (Halásztelek, Hungary); methanol (MeOH) from LGC Standards (Wesel, Germany); formic acid (FA) and LC–MS water from VWR Chemicals (Monroeville, PA, USA). Certified reference standards for all target analytes and their stable isotope-labelled internal standards (IS) were obtained from commercial suppliers (Toronto Research Chemicals, Toronto, ON, Canada; Sigma-Aldrich, St. Louis, MO, USA; Buchem B.V., Apeldoorn, The Netherlands), as described in the referenced methodological publications^30–32^.

Isotopically labelled IS were used for each analyte class to correct for matrix effects, ion suppression, and procedural variability. IS were added to samples at fixed concentrations prior to protein precipitation.

#### Calibration strategy and quality control

Eleven-point calibration curves were prepared in the appropriate matrix (water-based blank homogenate matrix) at concentrations spanning the expected physiological range for each analyte. Calibration standards and quality control (QC) samples at three concentration levels (low, medium, high) were freshly prepared for each analytical batch. Analyte concentrations were calculated using the ratio of the analyte peak area to the corresponding IS peak area, based on the slope and intercept of the linear regression calibration curve (no weighting applied). Acceptance criteria for each analytical batch required calibration R^2^ ≥ 0.99 and QC accuracy within ± 15% of nominal values (± 20% at the lower limit of quantification, LLOQ).

#### Chromatographic conditions

Chromatographic separation was performed on an Acquity UPLC system (Waters, Milford, MA, USA) using a reverse-phase C18 column (Acquity UPLC HSS T3, 5 µm, 2.1 × 150 mm; Waters) maintained at 15 °C. The mobile phase consisted of (A) 0.1% formic acid in water and (B) 0.1% formic acid in acetonitrile. A gradient elution program was used: 0–1.0 min, 3% B; 1.0–10.0 min, 3–23% B, 10.0–15.0 min, 23–95% B, 15.0–16.0 min, 95% B, 16.0–17.0 min, 95–3% B, 17–22.0 min, 3% B (re-equilibration). The flow rate was 0.4 mL/min and the injection volume was 10 µL. The total run time per sample was 22 min. Due to the wide polarity range of the analytes, a combined chromatographic approach covering both early-eluting polar metabolites (e.g., GSH, GSSG, NAD, PLP) and late-eluting hydrophobic indole metabolites (e.g., IPA, I3CA, pCS) was employed.

#### Mass spectrometry conditions and MRM transitions

A triple quadrupole mass spectrometer (QTRAP 5500, AB Sciex, Framingham, MA, USA) was operated in electrospray ionization (ESI) mode with both positive and negative polarity switching within a single run. Source parameters: curtain gas 30 psi, ion spray voltage + 5500 V (positive) / − 4500 V (negative), temperature 550 °C, ion source gas 1 and gas 2 each 50 psi. Data acquisition was performed in multiple reaction monitoring (MRM) mode. Each analyte was identified by (i) the specific MRM transition (precursor → product ion), (ii) the retention time within a ± 0.1 min window relative to the calibration standard, and (iii) the ion ratio (qualifier/quantifier MRM ≤ 30% deviation from the calibration standard where a second transition was available). Quantification was based on the quantifier transition only.

All MRM transitions, declustering potentials (DP), collision energies (CE), and chromatographic retention times are summarized in Supplementary Table S1.

#### Calculation of metabolite concentrations

Absolute metabolite concentrations in the striatal tissue samples were calculated using the following equation:

1 $${\mathrm{C}}\_{\mathrm{analyte}} = \left( {{\mathrm{Area}}\_{\mathrm{analyte}}/{\mathrm{Area}}\_{\mathrm{IS}}} \right) \times {\mathrm{C}}\_{\mathrm{IS}} \times ({\mathrm{1}}/{\mathrm{Response}}\_{\mathrm{factor}})$$

where C_IS is the known concentration of the internal standard added to the sample, and the response factor is derived from the slope of the calibration curve. Results are expressed as pmol/mg wet tissue weight, as calculated from the tissue weight measured prior to homogenization and the total volume of the reconstituted extract. All quantification was performed using Analyst software (version 1.7.3; AB Sciex).

### Statistical analysis

#### Softwares and packages

For the statistical analyses, R software (v. 4.5.1; R Foundation for Statistical Computing, Vienna, Austria) was used within the RStudio integrated development environment (v. 2025.05.1, PBC, Boston, MA, USA). The dataset analysis and visualizations were generated using the following R packages: tidyverse, mixOmics, ComplexHeatmap, EnhancedVolcano, circlize, ggplot2, stats and gridExtra.

#### Data preparation and biological classification

Metabolite concentration values were collected and cleaned by removing values that differed from the group mean by more than ± 3 standard deviations (SD) within each group independently for each metabolite^33^. In the final dataset, no values exceeded this threshold. One animal was excluded prior to analysis based on technical quality control criteria unrelated to the statistical outlier criterion. Where applicable, outliers were replaced with the median of the respective group to maintain data robustness, in line with previous studies^34–36^. To assess the impact of this procedure on the reported findings, a sensitivity analysis was conducted comparing results from the imputed and non-imputed datasets across all primary endpoints and key pairwise comparisons; no direction reversals were observed, and primary findings were robust across both datasets (Supplementary Table S2, sheet: ‘Sensitivity_analysis’). All metabolite data were subsequently standardized by Z-score transformation for cluster analysis.

From the concentration values, analyte ratios were calculated to serve as indicators of enzyme activities or metabolic balance states. Furthermore, metabolites and ratios for all 35 variables were categorized into groups according to their biological relevance (Neurotransmitters, Kynurenine pathway, Indole pathway/microbiome, Oxidative stress) for cluster analysis (Table 1).

**Table 1:** Summary of the quantified metabolites and the calculated ratios. Analyte ratios were used to assess enzyme activities or metabolic balance states.

Pathway	Analyte	Calculated ratios	Interpretation of ratios
Neurotransmitters	3MT, DA, DOPAC, HVA, MDOPA, MHPGS, TYR, TYRA		
Kynurenine pathway	TRP, KYN, KA, AA, XA, 3OHK, NAD, PLP	KYN/TRP	Indoleamine 2,3-dioxygenase (IDO), tryptophan 2,3-dioxygenase (TDO) enzyme activities
		KA/TRP	Indicator of flux toward neuroprotective branch
		KA/KYN	Kynurenine aminotransfeKase-1,2,3 (KATs) enyme activities
		AA/KYN	Kynureninase (KYNU) enzyme activity
		3OHK/KYN	Kynurenine monooxygenase (KMO) enyme activity
		XA/3OHK	Kynurenine aminotransferase-III (KATIII) enzyme activity
Indole pathway/microbiome	IAA, ILA, IPA, I3CA, INS, pCS	INS/TRP	Tryptophanase (TNA) enzyme activity
		IAA/TRP	Tryptophan 2-monooxygenase (TMO), tryptophan decarboxylase (TDO) , aromatic amino acid aminotransferase (ArAT) enzyme activities
		pCS/TYR	Indicator of gut microbial toxin production
Oxidative stress	GSH, GSSG	GSH/GSSG Oxidative Stress Index: 3OHK/(KA + AA + XA)	Indicator of oxidative stress balance

#### Oxidative stress index

To capture the balance between the neurotoxic and neuroprotective arms of the KP in a single integrative metric, the Oxidative Stress Index was defined as the ratio of the pro-oxidant metabolite 3OHK to the sum of neuroprotective metabolite concentrations (KA, AA, and XA) (Eq. 1):

1 *Oxidative Stress Index=(3OHK/(KA+AA+XA))*

A higher Oxidative Stress Index value indicates predominance of the neurotoxic branch and greater oxidative burden, whereas a lower Oxidative Stress Index reflects a shift towards neuroprotective metabolic flux^37–39^.

#### Multivariate analysis

For multivariate modelling, the supervised partial least squares discriminant analysis (PLS-DA) and the unsupervised principal component analysis (PCA) were performed. PCA and PLS-DA were applied solely for exploratory visualization of sample distribution and to identify overall variance patterns. These multivariate methods were not used to determine statistical significance between groups. Observed differences in the patterns were subsequently confirmed by univariate statistical analyses (Welch’s ANOVA with Games-Howell post-hoc test and Welch’s two-sample *t*-test), ensuring that group differences did not originate from model overfitting.

To assess the validity and predictive performance of the PLS-DA models, internal validation was performed using sevenfold cross-validation, and model fit was evaluated by reporting R^2^Y (goodness of fit) and Q^2^ (predictive ability) metrics. To guard against overfitting, permutation testing was conducted with 1000 permutations; a model was considered valid if the Q^2^ values of the permuted models were lower than that of the original model, and if the intercept of the permuted Q^2^ distribution was below zero. To identify the most important metabolites contributing to group separation, variable importance in projection (VIP) scores were extracted from the PLS-DA models. VIP values were summarized in ranked tables and visualized as bar plots (top 20 features).

#### Cluster analysis

For the cluster analysis, all metabolite concentrations were first standardized to Z-scores per metabolite across all samples (mean = 0, SD = 1) prior to analysis. Mean standardized values within biological pathway categories (Table 1) were subsequently calculated for each sample and visualized in sample- and group-level heatmaps. To further illustrate differences between the groups, sample similarity patterns were investigated by hierarchical clustering using Euclidean distance and Ward’s linkage. Global patterns across metabolites and pathways were visualized as heatmaps applying consistent Z-score color scaling. Correlation structures among metabolites were assessed using Pearson’s (r) correlation coefficients and visualized as clustered correlation heatmaps.

#### Univariate analysis

Differences between groups were evaluated using Welch’s ANOVA followed by Games–Howell post-hoc test where the variances were unequal. One-way ANOVA, followed by Tukey’s HSD post-hoc test was used for normally distributed data with equal variances, and the Kruskal–Wallis test for non-parametric data. Welch’s two-sample *t*-test was applied for pairwise comparisons used in volcano plot analysis. To account for multiple testing, *p*-values were corrected using the Benjamini–Hochberg false discovery rate (FDR) method, applied within each pairwise comparison independently across all metabolites tested simultaneously. Adjusted *p*-values < 0.05 were considered statistically significant. Box plots and volcano plots were created for visualization. In the volcano plots Log2 fold changes were calculated for all metabolites; metabolites with |log2FC|≥ 1 and FDR adjusted *p* < 0.05 were considered statistically significant.

#### Effect size estimation

Given the relatively small and unequal group sizes inherent to the experimental design, Cohen’s d was calculated for all pairwise comparisons as a standardized measure of effect size, using pooled standard deviation. Effect sizes were interpreted as negligible (|d|< 0.2), small (0.2 ≤|d|< 0.5), medium (0.5 ≤|d|< 0.8), or large (|d|≥ 0.8). To further assess the robustness of the findings, bootstrap 95% percentile confidence intervals (CI) for Cohen’s d were estimated using 10,000 resampling iterations (seed = 42) for key pairwise comparisons. A 95% CI excluding zero was considered indicative of a robust effect independent of sample size. Complete effect size results with bootstrap CI are provided in Supplementary Table S3 (sheet: ‘Effect_sizes_95CI’).

## Results

### Identification of key alterations by multivariate analysis

**Fig. 2:**
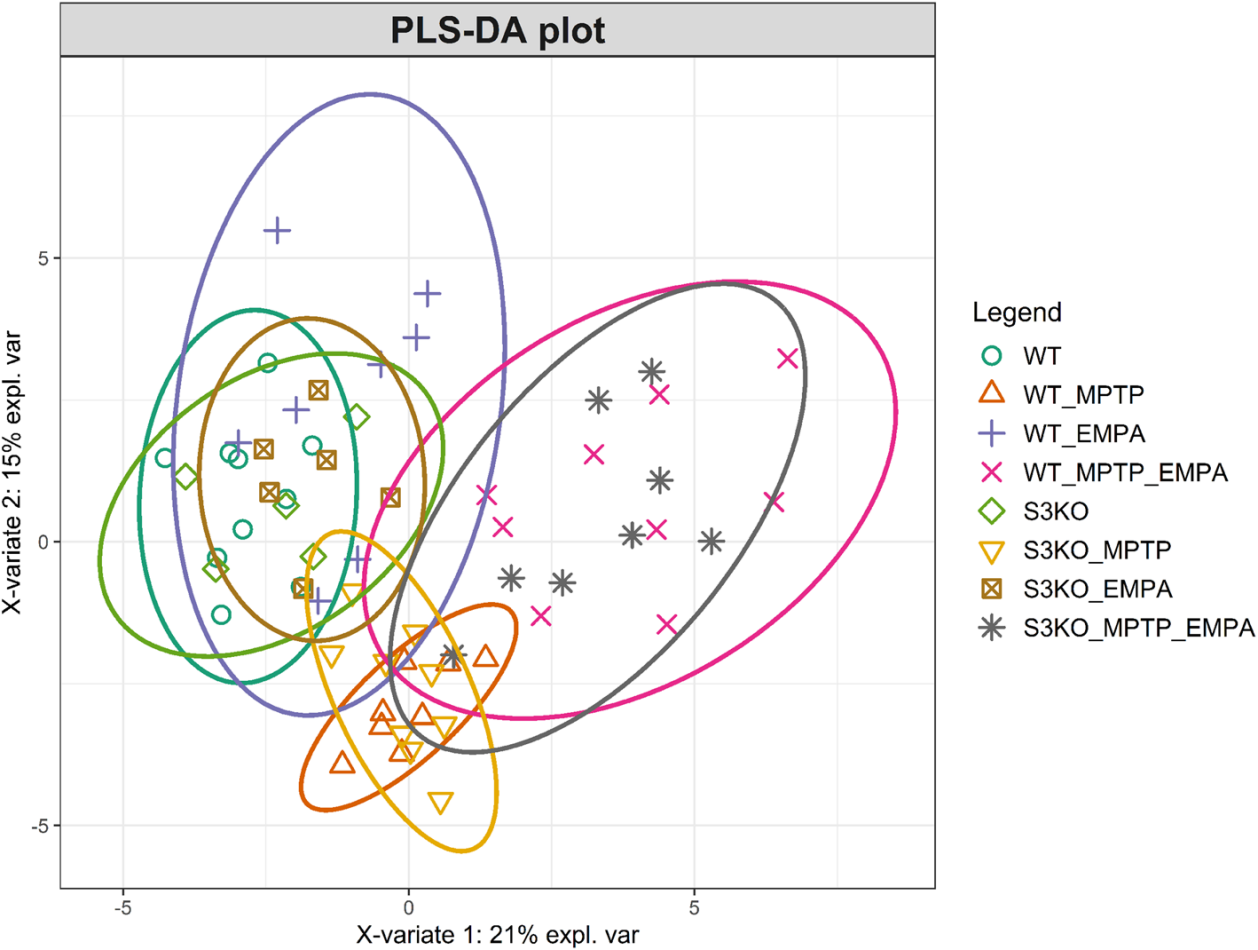
PLS-DA score plot of all eight experimental groups based on 35 striatal metabolites (three components). Model validation: sevenfold cross-validation with 10 repeats yielded R^2^X = 0.45, R^2^Y = 0.43, Q^2^Y = 0.24 (R^2^Y − Q^2^Y = 0.19). Permutation testing (*n* = 1000) confirmed model validity: both R^2^Y (*p* = 0.023) and Q^2^Y (*p* < 0.001) significantly exceeded permuted values. Ellipses represent 95% confidence regions.

**Fig. 3:**
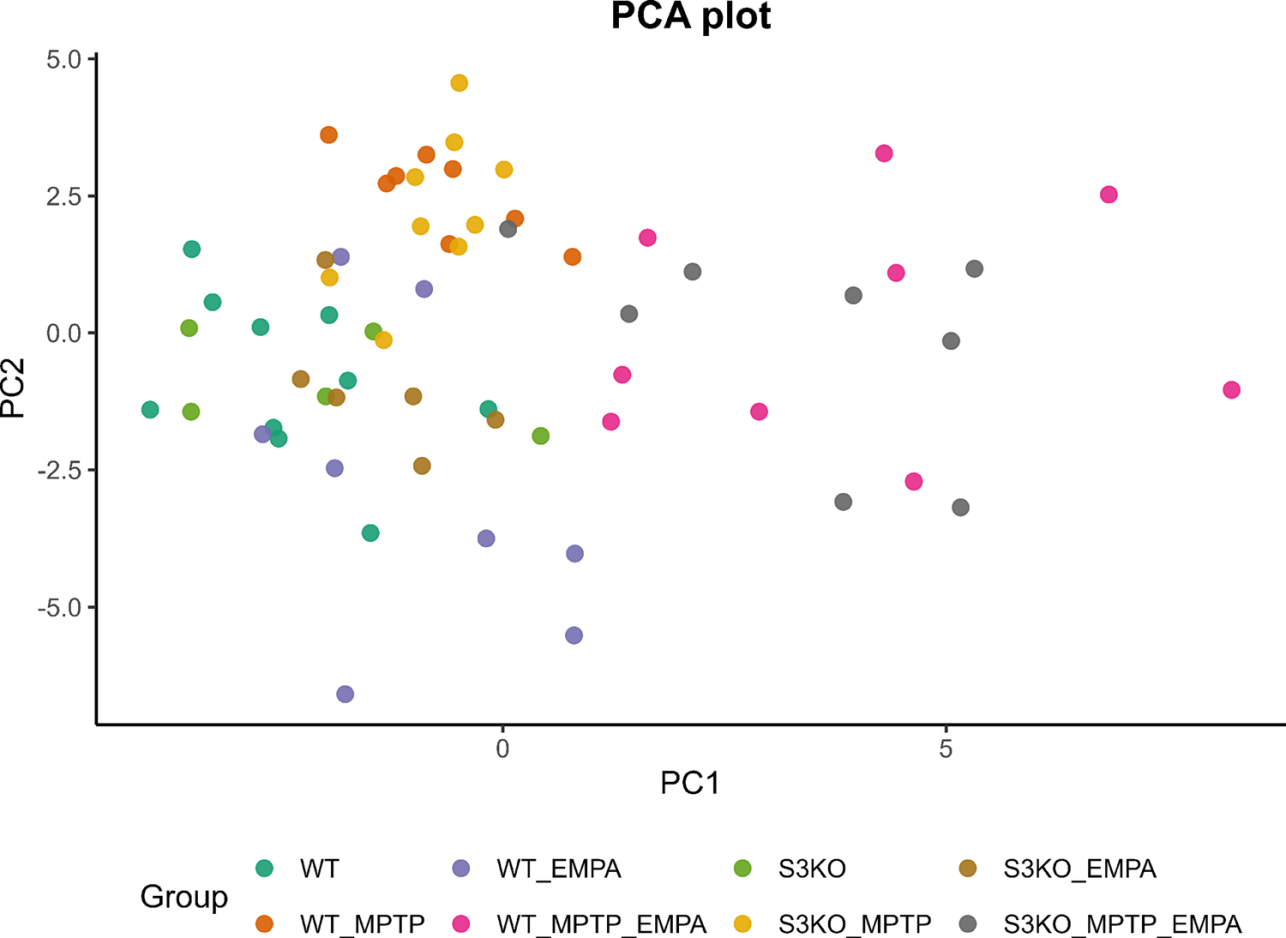
The PCA plot illustrates similarities and differences among samples based on their metabolic profiles.

**Fig. 4:**
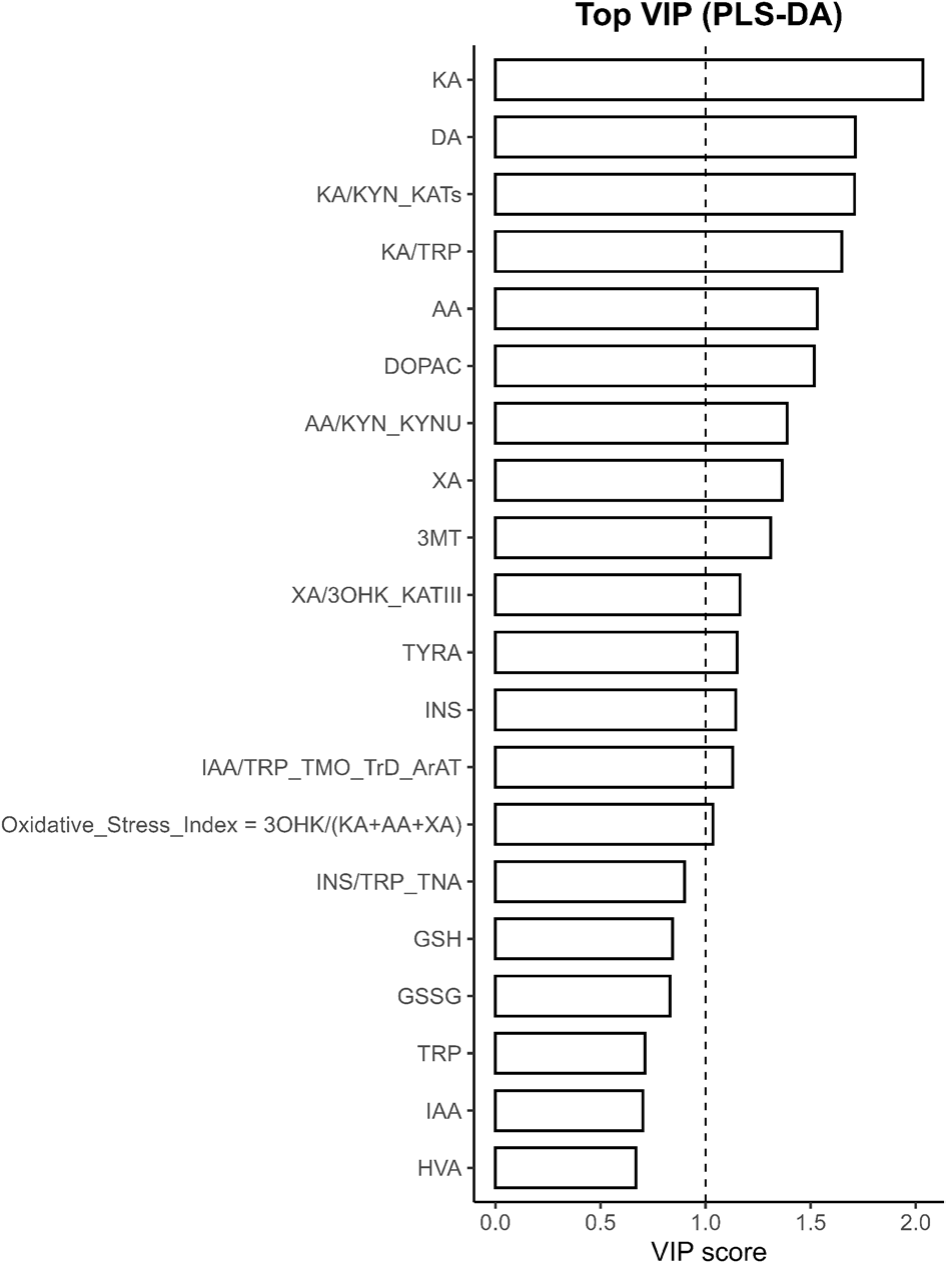
VIP score list represents the top 20 variables which refers to the highest variability between the groups.

Multivariate statistical analysis was performed on all 35 metabolic variables across 8 groups to assess how the samples separate from each other based on their metabolic profiles. PLS-DA and PCA can reduce complex, high-dimensional datasets into new components that capture the most dominant patterns, enabling the visualization of similarities and separations among samples. Accordingly, PCA and PLS-DA were used to explore global patterns rather than to statistically test for significant differences between groups. The PLS-DA model (3 components) demonstrated acceptable fit and predictive performance, as confirmed by internal validation: sevenfold cross-validation with 10 repeats yielded R^2^X = 0.45, R^2^Y = 0.43, and Q^2^Y = 0.24. The low difference between R^2^Y and Q^2^Y (Δ = 0.19) indicates that the model does not suffer from substantial overfitting. Permutation testing (*n* = 1000 permutations) confirmed model validity, as both R^2^Y (*p* = 0.023) and Q^2^Y (*p* < 0.001) significantly exceeded values obtained from models built on randomly permuted class labels, demonstrating that the observed group separation reflects genuine metabolic differences rather than chance. In the PLS-DA plot, samples with similar metabolic patterns cluster together within the same confidence ellipse **(**Fig. 2**)**. In contrast, the PCA model identifies variation solely based on the total variance of the analytes, without using any group information (Fig. 3). The analysis revealed distinctions between the 8 groups based on their spatial distribution. The MPTP + EMPA-treated animals (WT_MPTP_EMPA, S3KO_MPTP_EMPA) strongly differed from all the other groups. The ellipses of the WT, S3KO, WT_EMPA and S3KO_EMPA groups overlapped, suggesting a similar metabolite pattern (Fig. 2). In addition, no differences were detected in the metabolic pattern of WT_MPTP and S3KO_MPTP groups. In order to explore which variables are responsible for the variance between groups, VIP scoring was performed (Fig. 4). KA had the highest VIP score, followed by the AA, and XA from the KP and the calculated ratios (e.g., KYNU, KATs). The second highest score was found for dopamine (DA), which supports effectivity of the MPTP treatment to induce dopaminergic neuronal dysfunction. The all-VIP score can be found in the Supplementary Table S4. The results of the multivariate statistical analysis showed different metabolic patterns in MPTP + EMPA-treated groups compared to other groups, and the VIP scoring highlighted an altered TRP metabolism. Univariate statistical analyses were additionally performed to evaluate significant differences in the metabolic levels between treatments.

#### Pathway-Level clustering from groups to samples

**Fig. 5:**
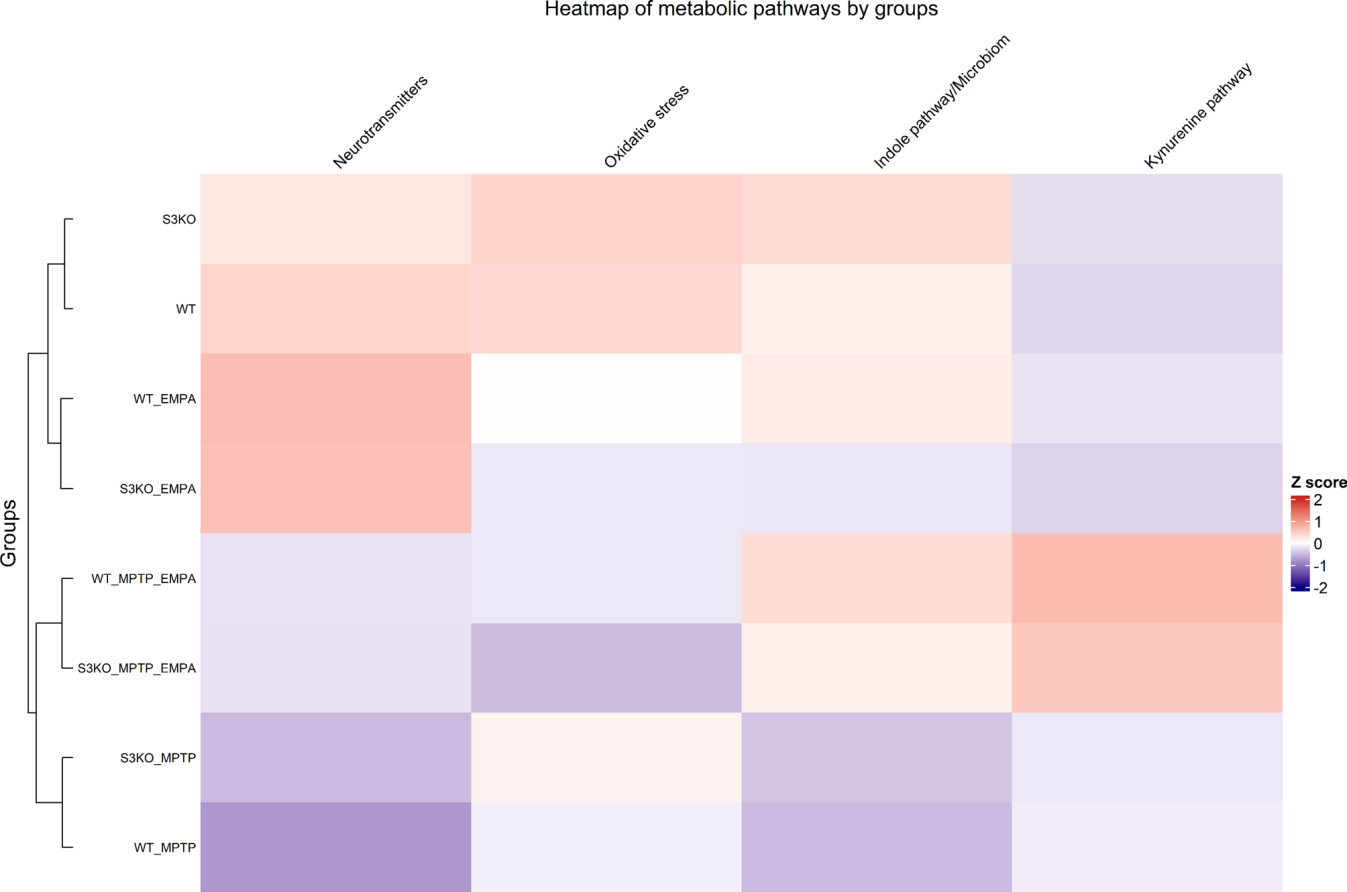
Heatmap of metabolic pathways among groups. Groups with similar metabolite profiles were clustered together using hierarchical clustering (Euclidean distance, Ward’s linkage). Values represent mean Z-scores across all metabolites within each biological pathway category (Neurotransmitters, Oxidative stress, Indole pathway/Microbiome, Kynurenine pathway), calculated per metabolite across all samples (mean = 0, SD = 1). Red shading indicates Z-scores higher than the overall mean, whereas purple shading indicates Z-scores lower than the overall mean metabolite concentrations.

**Fig. 6:**
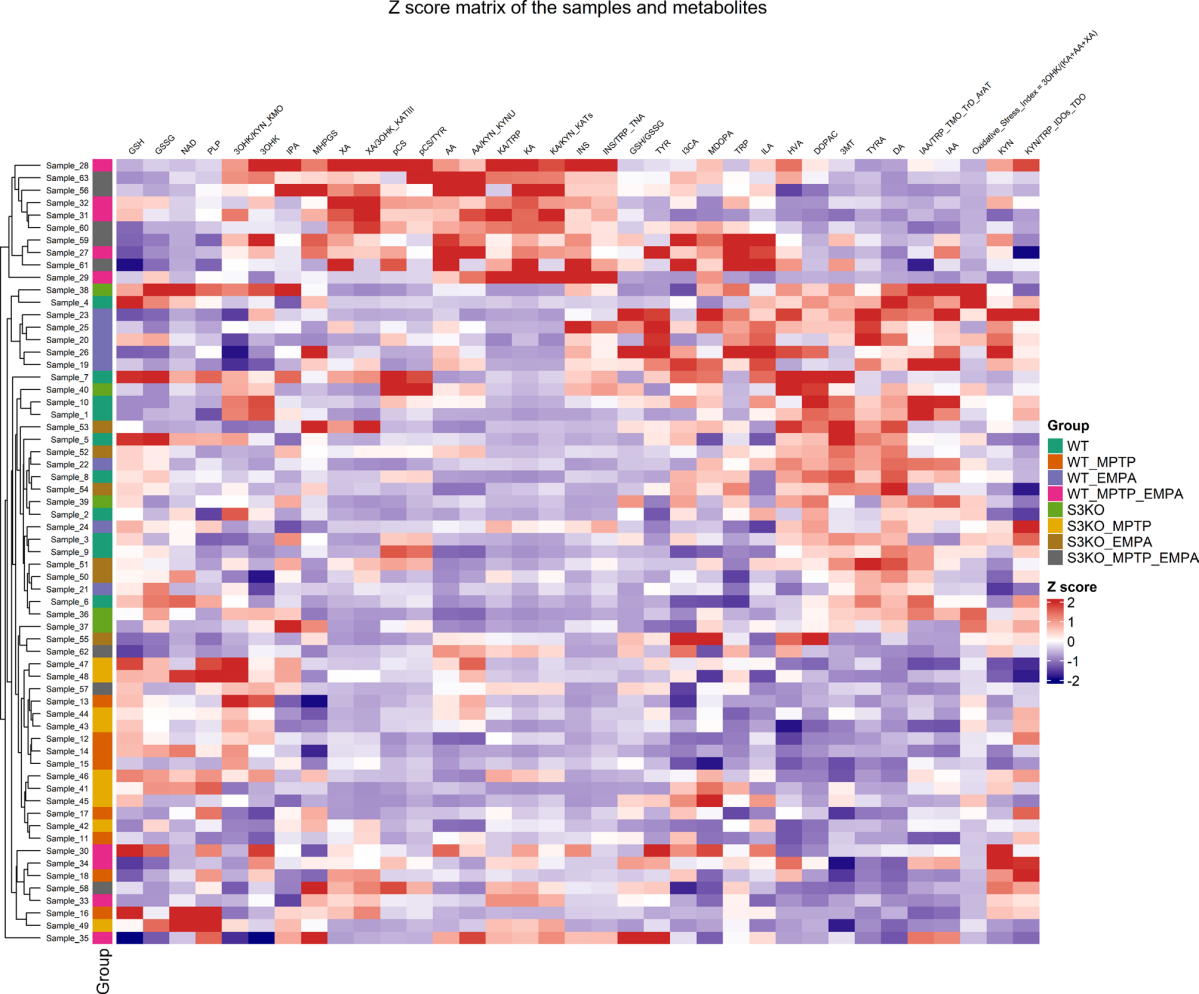
Z-score matrix of individual samples, metabolites and ratios. Values represent Z-scores calculated per metabolite across all samples (mean = 0, SD = 1). Hierarchical clustering was applied to both rows (samples) and columns (metabolites) using Euclidean distance and Ward’s linkage. Red shading indicates Z-scores above the mean, purple shading indicates Z-scores below the mean.

**Fig. 7:**
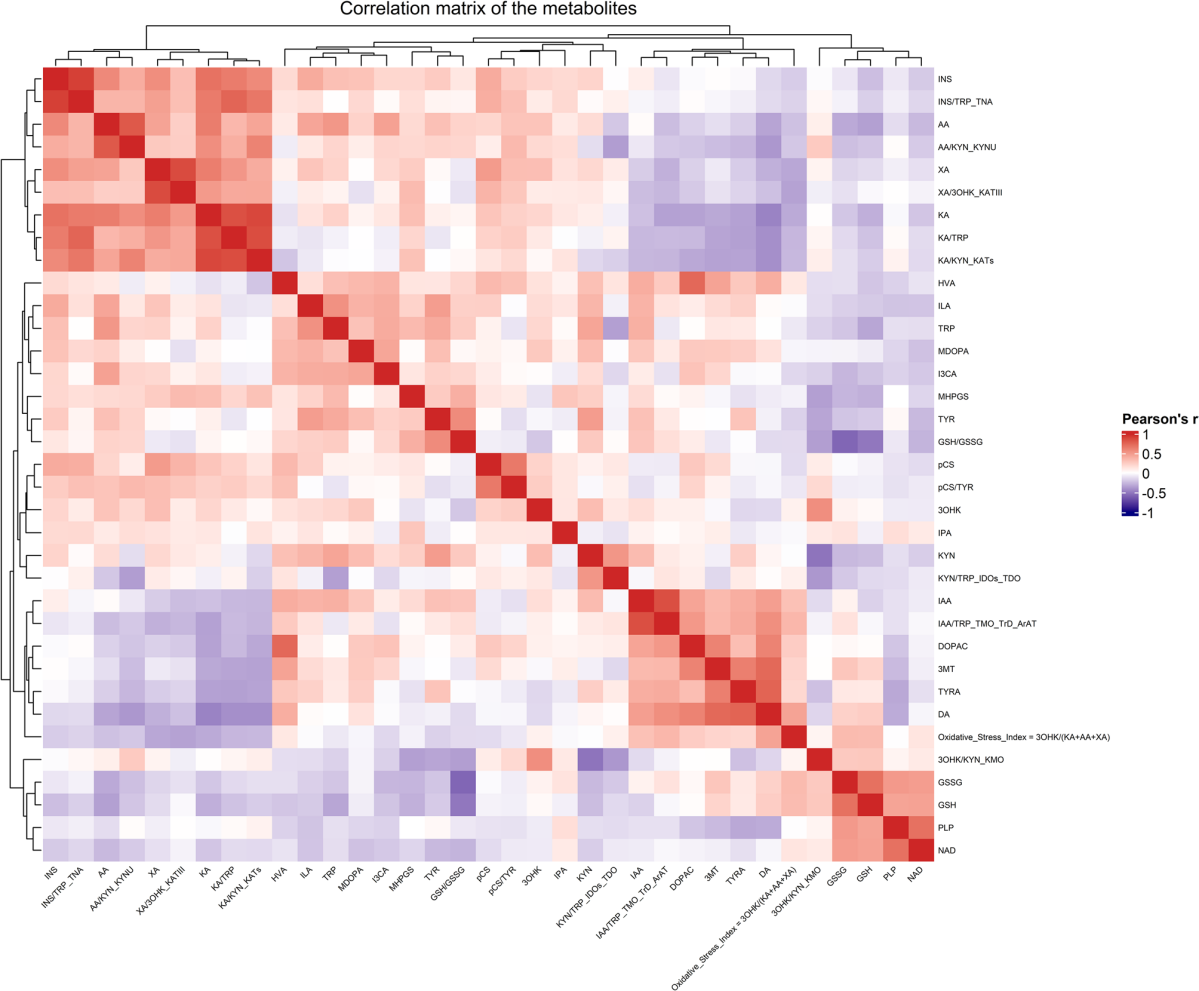
The heatmap represents Pearson correlation coefficients (r) between metabolites and ratios, calculated across all samples. This correlation matrix shows the association between each pair of 35 variables. Red and purple shading indicate positive and negative correlations, respectively. The KP-related metabolites and ratios showed strong positive correlation with each other. Similarly, neurotransmitters correlated positively with each other. Negative correlations were observed between GSH/GSSG and GSSG, as well as between KYN and 3OHK/KYN_KMO.

Clustered heatmap analysis was applied to investigate the differences between the groups based on their metabolic pattern. For this purpose the concentration values of all metabolites and their ratios were transformed to Z-score in all samples. The 35 variables were divided into 4 metabolic pathways as described in the Methods section. The S3KO, WT and S3KO_EMPA, WT_EMPA groups fell into to the same cluster since they had similar metabolic patterns (Fig. 5), showing that EMPA treatment alone did not cause significant changes compared to the control groups. The MPTP-treated groups WT_MPTP and S3KO_MPTP fell into a common cluster due to decreased levels of dopaminergic neurotransmitters. In the neurotransmitters column the Z-scores in the MPTP-treated groups (WT_MPTP, S3KO_MPTP) were lower than the mean. In the MPTP + EMPA-treated groups Z-scores of the KP metabolites were substantially elevated, while markers of the oxidative stress (GSH, GSSG, GSH/GSSG, Oxidative Stress Index) were lower. To examine the cluster analysis in more detail, a Z-score matrix was created on the samples and metabolites levels (Fig. 6). The figure highlights how samples segregate based on their metabolic patterns, illustrating both similarities within groups and differences between groups. Overall, the Z-score representation facilitates the identification of metabolic trends and potential biomarkers by standardizing metabolite levels among samples. Correlation coefficients for each pair of the 35 variables (metabolites and ratios) are represented in Fig. 7. Strongest correlations were noted between the kynurenines and the ratios that represent enzyme activities. In addition, positive correlation was observed in the case of dopaminergic system components such as DA, HVA and DOPAC. Notable negative correlation was observed between the GSSG and the GSH/GSSG.

#### Identification of significantly altered metabolites

**Fig. 8:**
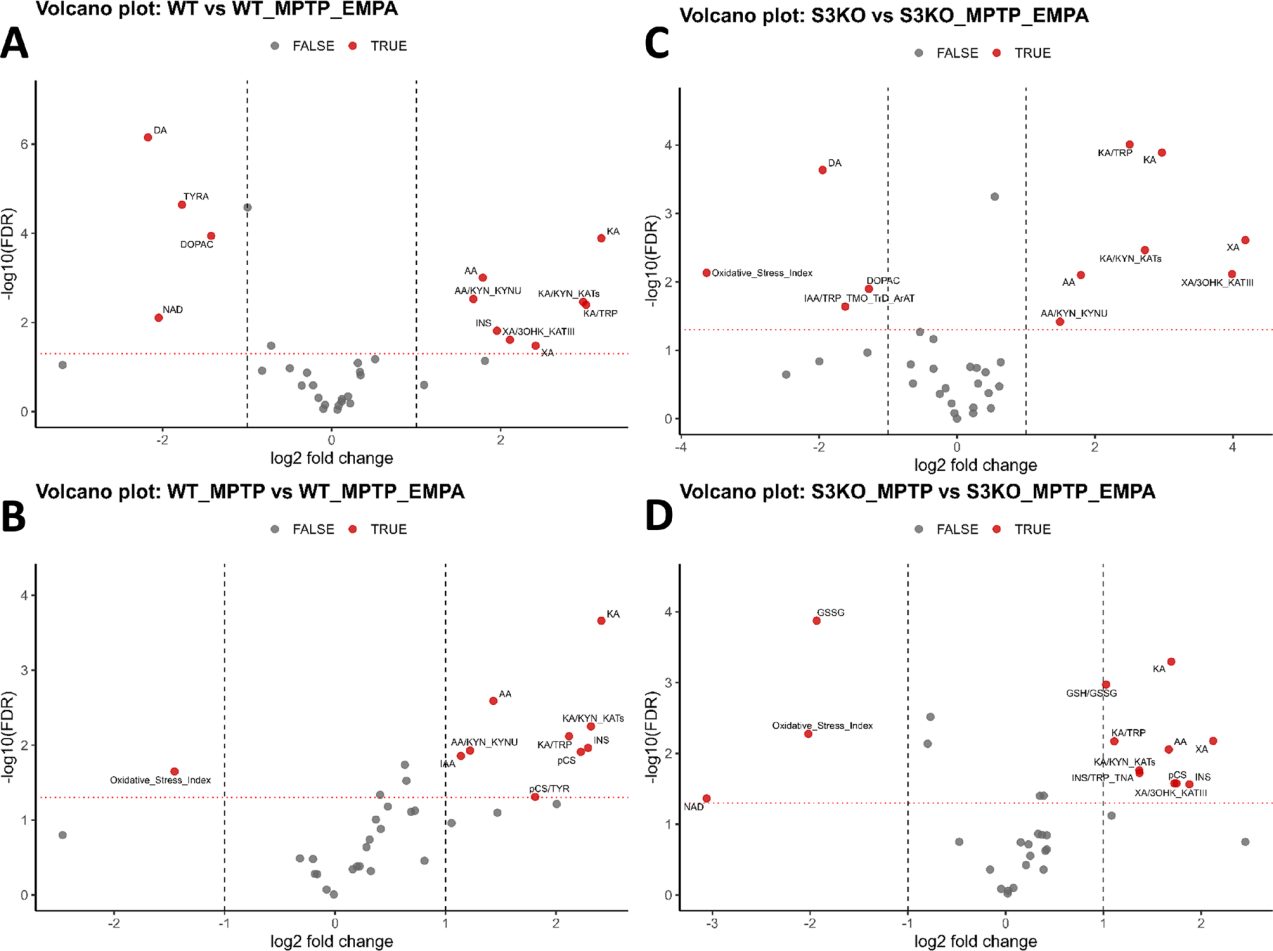
Volcano plots displaying significantly different metabolites between **(A)** WT vs. WT_MPTP_EMPA, **(B)** WT_MPTP vs. WT_MPTP_EMPA, **(C)** S3KO vs. S3KO_MPTP_EMPA, **(D)** S3KO_MPTP vs. S3KO_MPTP_EMPA. The x-axis represents the log₂ fold change (log₂FC), indicating the magnitude and direction of the difference. The y-axis shows the statistical significance, i.e., Welch’s two-sample* t*-test. *p*-values were adjusted by the Benjamini–Hochberg FDR correction (− log₁₀ FDR). Metabolites that simultaneously pass the log₂FC (|log₂FC|≥ 1) and FDR (*p* < 0.05) thresholds are considered significantly different between groups.

The multivariate and cluster analysis results suggested that the MPTP + EMPA-treated groups had similar metabolic patterns which were different from the other groups predominantly in the KP metabolites. To identify significant pairwise differences between groups, Welch’s two-sample *t*-test was applied for all pairwise comparisons used in the volcano plot analysis. Welch’s ANOVA with Games-Howell post-hoc test was applied separately for overall multi-group effect testing. Raw *p*-values from each pairwise Welch’s *t*-test were corrected for multiple testing using the Benjamini–Hochberg False Discovery Rate (FDR) method, applied within each pairwise comparison independently across all 35 metabolites simultaneously. A metabolite was considered statistically significant if it fulfilled both criteria: FDR-adjusted *p*-value < 0.05 and |log₂FC|> 1. Volcano plots were created for pairwise comparisons to depict significantly increased and decreased metabolites simultaneously (Fig. 8). The plots show comparisons of MPTP + EMPA-treated animals (WT_MPTP_EMPA, S3KO_MPTP_EMPA) with the controls (WT, S3KO) and with the MPTP-treated groups (WT_MPTP, S3KO_MPTP), respectively. A significant increase was observed in KA, XA and AA levels and the respective enzymatic activity ratios (AA/KYN_KYNU, KA/KYN_KATs, XA/3OHK_KATIII, KA/TRP) in the MPTP + EMPA-treated groups compared with all other groups. Furthermore, the magnitude of fold-change differences in the S3KO_MPTP_EMPA vs S3KO comparison (observed |log₂FC| range: 2–4) was notably more pronounced than in the corresponding WT comparison, indicating a genotype-dependent amplification of the EMPA effect on KP metabolites (Fig. 8C–D). Along with the decrease detected in Oxidative Stress Index, the decline of GSSG was also observed in MPTP + EMPA-treated animals (S3KO vs. S3KO_MPTP_EMPA, S3KO_MPTP vs. S3KO_MPTP_EMPA) when compared to S3KO groups (Fig. 8C–D). Furthermore, the decrease of DA and DOPAC in MPTP + EMPA treated animals could also be observed in contrast to those not treated with MPTP (Fig. 8A–D. Further volcano plots on group comparisons are shown in Supplementary Figure S1 and the detailed statistics including FDR-adjusted *p*-values and Cohen’s d for all comparisons shown in Supplementary Table S5.

**Fig. 9:**
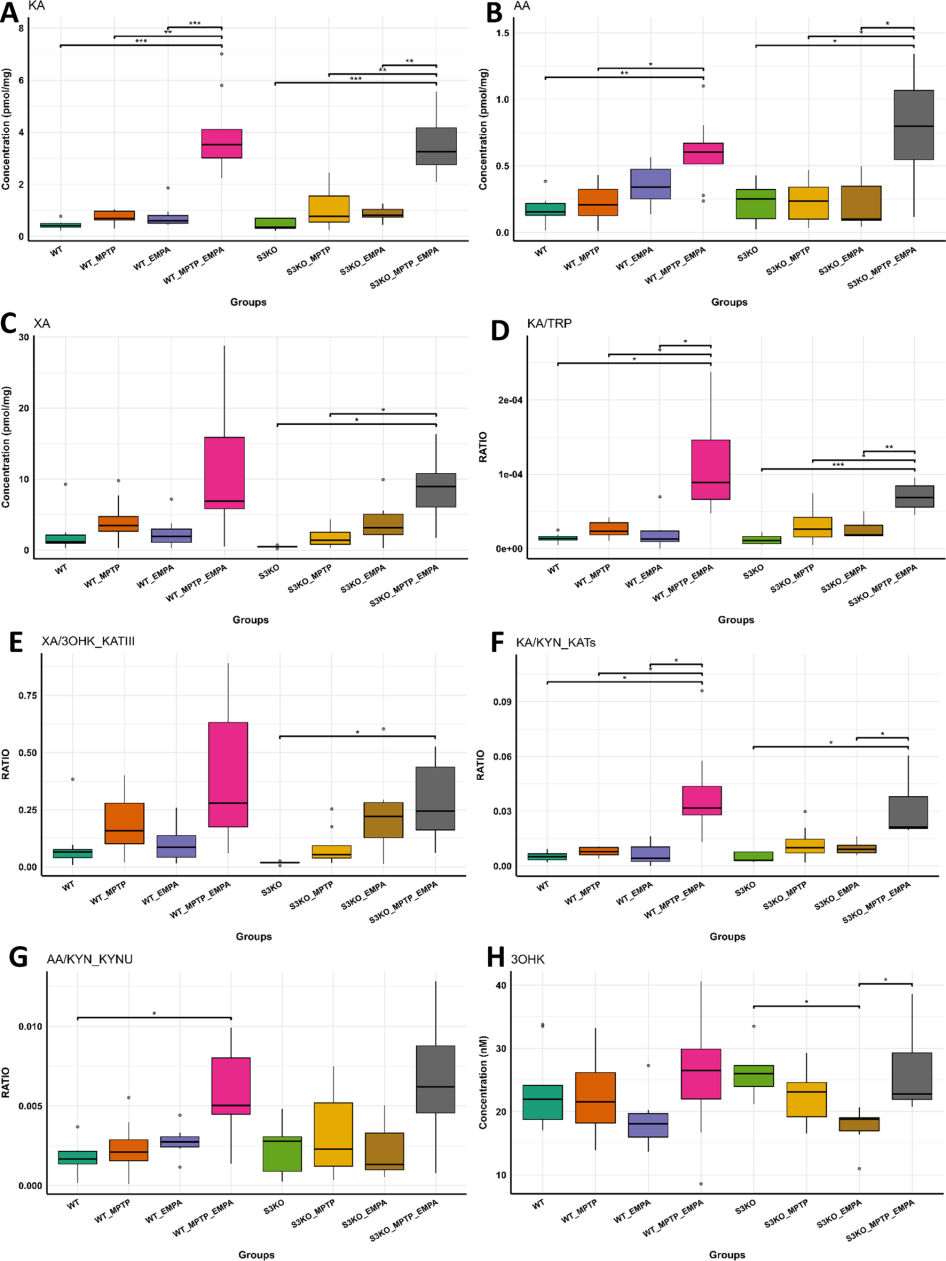
Changes in KP metabolite levels, ratios in response to different treatments (MPTP; EMPA; MPTP + EMPA) and in control groups (WT, S3KO). The boxplots display the within-group distribution of metabolites and ratios by showing the median, interquartile range (IQR), and outliers. A significant increase in KA **(A)**, AA **(B)**, XA **(C)**, KA/TRP **(D)**, XA/3OHK_KATIII **(E)**, KA/KYN_KATs **(F)** and AA/KYN_KYNU **(G)** levels were observed in the MPTP + EMPA-treated groups (WT_MPTP_EMPA, S3KO_MPTP_EMPA) compared to the EMPA, MPTP treated-, and control groups. In the 3OHK **(H)** a significant decrease was observed in the EMPA treated S3KO groups (S3KO_EMPA; S3KO_MPTP_EMPA) compared to the S3KO control group. (Welch’s ANOVA, Games-Howell post-hoc; test; *: *p* < 0.05; **: *p* < 0.01; ***: *p* < 0.001).

**Fig. 10:**
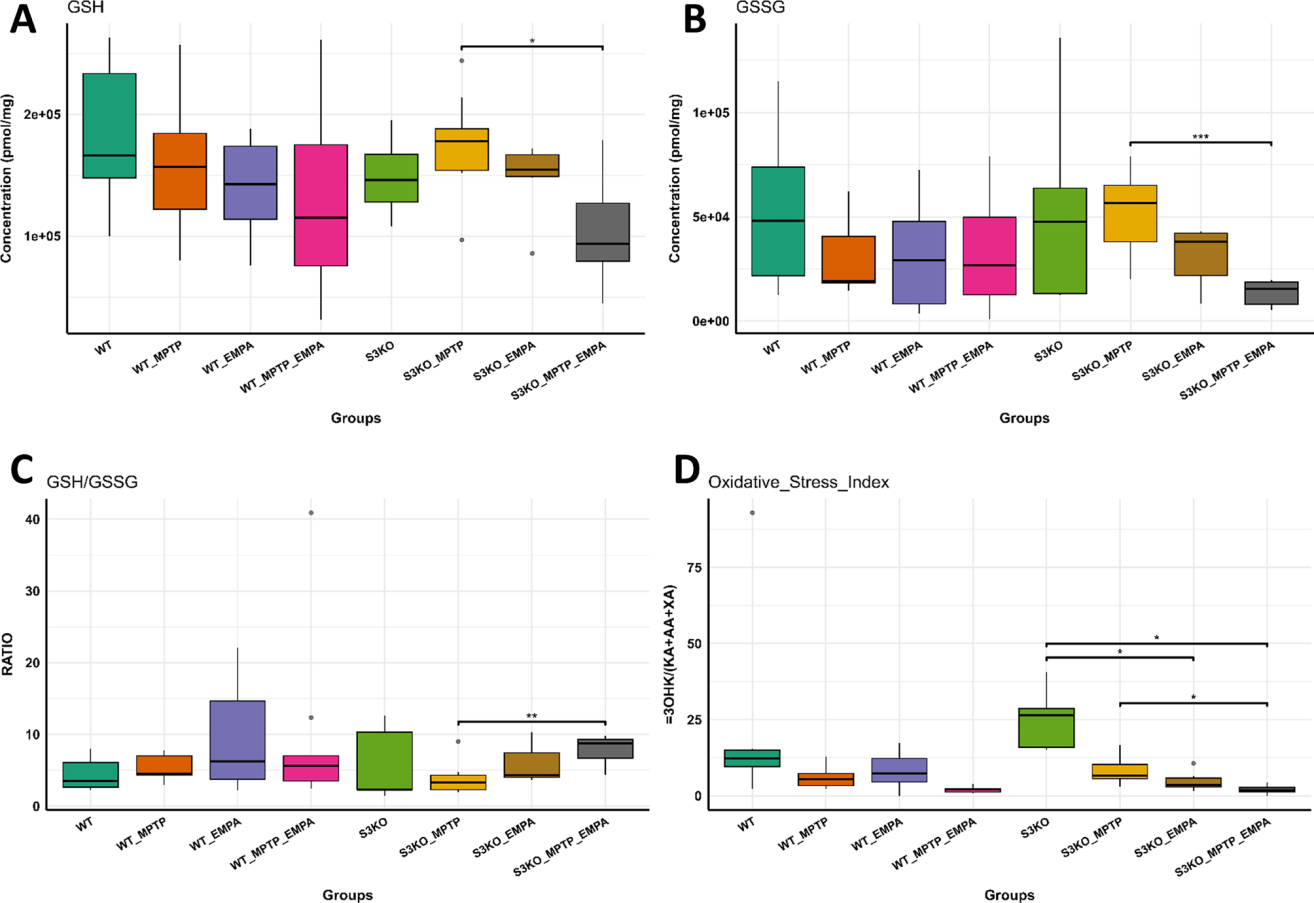
Changes in glutathione system and Oxidative_Stress_Index in response to different treatments (MPTP; EMPA; MPTP + EMPA) and in control groups (WT, S3KO). The boxplots display the within-group distribution of metabolites and ratios by showing the median, interquartile range (IQR), and outliers. The GSH **(A)** and GSSG **(B)** were significantly decreased in the S3KO_MPTP_EMPA group, while the GSH/GSSG ratio **(C)** significantly increased compared to the MPTP treated S3KO group. In the Oxidative_Stress_Index **(D)** a significant decrease was observed in the EMPA treated S3KO groups (S3KO_EMPA; S3KO_MPTP_EMPA) compared to the S3KO control group. (Welch’s ANOVA, Games-Howell post-hoc; test; *: *p* < 0.05; **: *p* < 0.01; ***: *p* < 0.001).

Boxplots representing the most relevant KP metabolites and ratios showing significant differences—including KA, XA, AA, 3-OHK, KATs, KATIII, KA/TRP, and AA/KYN (KYNU)—are shown in Fig. 9. Glutathione-related parameters (GSH, GSSG, GSH/GSSG ratio) and the Oxidative Stress Index are presented in Fig. 10. All *p*-values are summarized in Supplementary Table S6.

Neuroprotective kynurenine metabolites (KA, XA, AA) and the corresponding enzymatic activity ratios were significantly increased in both WT and S3KO MPTP + EMPA-treated groups compared to their respective MPTP-only, EMPA-only, and untreated control groups, suggesting that EMPA augments the neuroprotective arm of the KP irrespective of SIRT3 genotype.

In the S3KO group, EMPA treatment—both alone and in combination with MPTP—significantly reduced the Oxidative Stress Index, indicating a shift towards a less neurotoxic kynurenine metabolic profile. Notably, EMPA treatment alone significantly decreased 3OHK levels in the S3KO EMPA group, suggesting that SIRT3 deficiency renders the KP particularly sensitive to EMPA-mediated metabolic modulation.

Regarding glutathione redox status, both GSH and GSSG concentrations were decreased in the S3KO MPTP + EMPA group compared to S3KO MPTP animals. Critically, GSSG declined to a proportionally greater extent than GSH, resulting in a significantly elevated GSH/GSSG ratio in the S3KO MPTP + EMPA group. This pattern is consistent with a reduction in overall glutathione turnover driven by diminished oxidative load, rather than a depletion of the total glutathione pool, and reflects an improvement in cellular redox homeostasis. This interpretation is further supported by the parallel reduction of the Oxidative Stress Index in the same group, providing convergent evidence from two biochemically independent pathways—the glutathione redox system and the KP—for EMPA-mediated attenuation of oxidative stress in the SIRT3-deficient, MPTP-lesioned background. Comparisons of remaining metabolites and ratios are provided in Supplementary Figure S2.

### Effect sizes and confidence intervals for primary endpoints

Supplementary Table S3 summarizes the Cohen’s d effect sizes with bootstrap 95% CI for the primary endpoints across the four key pairwise comparisons. The primary KP endpoints (KA, AA, XA) and Oxidative Stress Index demonstrated large, robust effect sizes with confidence intervals excluding zero across all comparisons, confirming the biological relevance of the observed changes despite the relatively small group sizes. Glutathione redox parameters showed robust effects specifically in the S3KO group comparisons, while EMPA did not produce robust effects on dopaminergic markers in MPTP-treated animals.

## Discussion

### Summary of findings

The present study aimed to investigate metabolic changes in response to MPTP- and MPTP + EMPA treatments in WT and S3KO animals. For this purpose neurologically relevant metabolites were quantified using targeted UHPLC–MS/MS method, focused on KP metabolites and oxidative stress markers. A multi-level statistical strategy was employed, proceeding from global multivariate pattern recognition (PLS-DA, hierarchical clustering) through pathway-level visualization (Z-Score heatmaps) to individual metabolite comparisons (volcano plots, univariate testing with FDR correction, box plots). The results revealed that EMPA treatment modulated the levels of neuroprotective KP metabolites and reduced the oxidative stress in the MPTP-induced PD model, consistent with a potential neuroprotective metabolic profile. The underlying mechanisms of EMPA-induced metabolic alterations are discussed below.

### MPTP effectiveness for the PD model

MPTP is the most commonly used neurotoxin to induce PD in animal models^20^. In the MPTP-treated groups, concentrations of dopaminergic neurotransmitters were significantly (*p* < 0.05) decreased compared to the control groups, indicating an effective induction of PD. This effect is further confirmed by the levels of DA and DOPAC as shown in Supplementary Figure S2.

### EMPA effects on the neuroprotective kynurenines

**Fig. 11:**
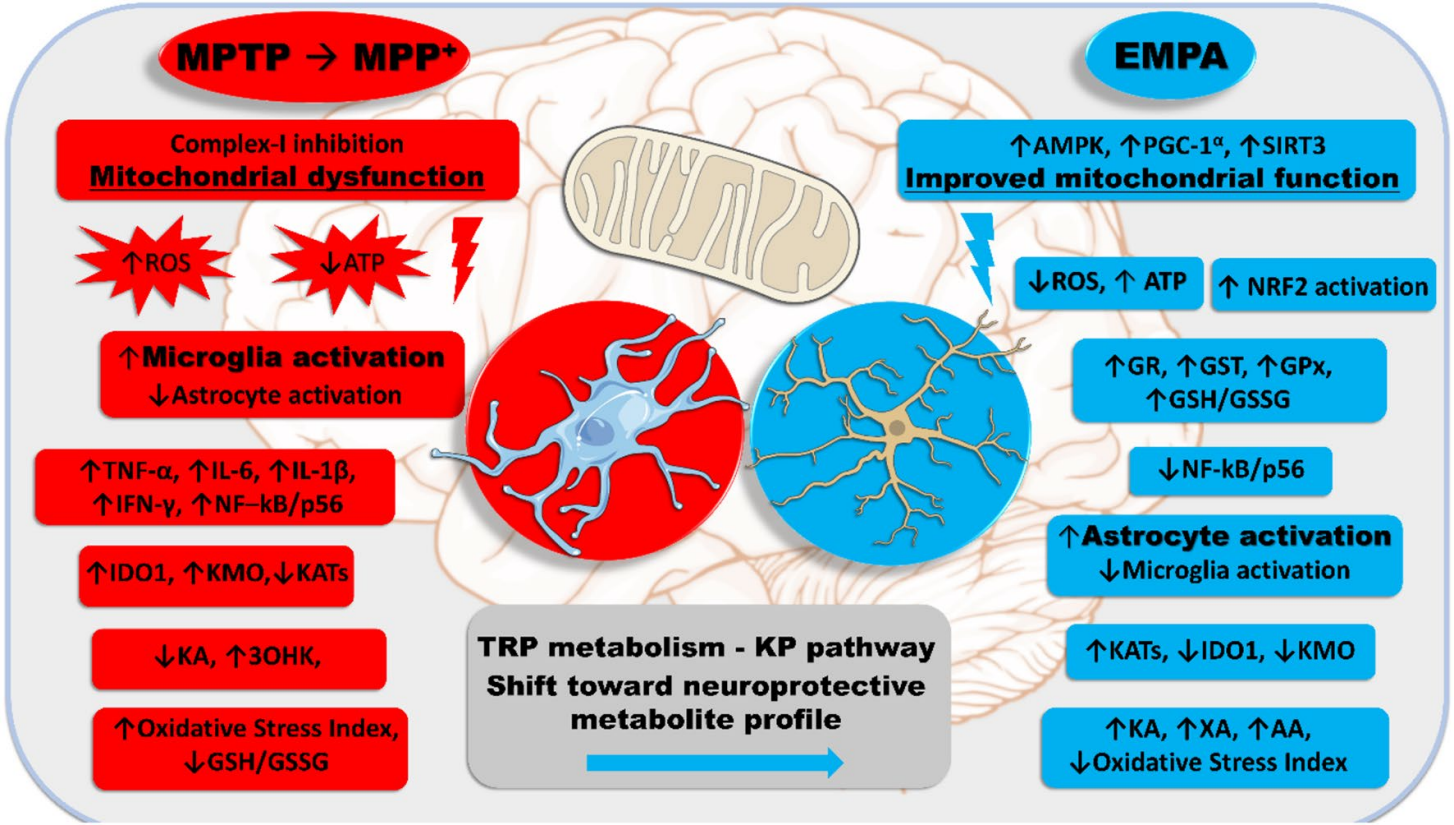
Schematic diagram of the proposed effects of MPTP and EMPA on KP metabolism and oxidative stress in the striatum, based on the metabolite-level changes observed in the present study and mechanistic frameworks derived from published literature. Left panel (red): MPTP-induced pathological changes associated with neurotoxic KP flux and oxidative burden. Right panel (blue): EMPA-associated metabolic changes consistent with a shift towards neuroprotective KP metabolites and improved redox homeostasis.

Previous studies have highlighted the neuroprotective effects of EMPA in neurodegenerative disorders such as Alzheimer’s and Parkinson’s disease models^27–29^. Prophylactic EMPA administration was shown to reduce PD risk and protect against PD-related cardiac dysfunction^40,41^. Additionally, a previous study on its effects on the tryptophan metabolism reported altered kynurenine levels in myocardium^42^. There is growing evidence that EMPA has effects additional to the inhibition of the SGLT2. Indirect effects exerted by EMPA may be attributed to the diverse neuronal metabolite expression patterns. Based on this body of evidence, the mechanistic explanations presented below are derived from published literature and are proposed as a framework consistent with—but not directly demonstrated by—our metabolomics data, which are schematically presented in Fig. 11.

Several studies have reported reduced KA levels in the brain samples of PD patients in post-mortem examinations^43^. In this study only the MPTP treatment did not affect significantly the KP metabolites compared to the control groups. Our data demonstrate that the neuroprotective kynurenines (KA, AA, XA) concentrations were significantly increased in MPTP + EMPA-treated animals in both WT and S3KO groups, indicating that EMPA selectively augments the neuroprotective branch of the KP in the context of MPTP-induced neurodegeneration. To interpret this finding mechanistically, the immunomodulatory role of kynurenines and the cell-type-specific expression of KP enzymes must be considered—both of which are established in the published literature^44^. During inflammatory responses, IDO expression is regulated by inflammatory cytokines (IFN-*γ*, TNF-α) and transcription factors (NF-κB, p65), resulting an increased production of kynurenines^45,46^. Furthermore, astrocytes and microglia exhibit fundamentally different KP enzyme expression profiles: under physiological conditions, KYN-to-KA conversion preferentially occurs in astrocytes due to high KAT activity, whereas microglia predominantly metabolize KYN towards 3OHK and QA via KMO^47^. Based on these published findings, we propose that the selective elevation of KA/TRP—without a corresponding change in KYN/TRP observed in our data—reflects enhanced astrocytic KAT activity rather than a generalized shift in IDO/TDO-driven tryptophan catabolism.

Additionally, published evidence indicates that EMPA activates Nuclear Factor erythroid 2-related factor 2 (NRF2) via the 5’-adenosine monophosphate (AMP)-activated protein kinase (AMPK) pathway, which enhances the production of anti-inflammatory and antioxidant molecules in astrocytes, thereby supporting the production of neuroprotective kynurenines such as KA^48^. Consistent with this mechanism, our data show a concurrent reduction in the Oxidative Stress Index (3OHK/(KA + AA + XA)) in EMPA-treated S3KO animals, suggesting that the improvement in the redox environment may have reduced pro-oxidant 3-OHK formation while facilitating KA production—an interpretation supported by, but not directly demonstrated in, the present dataset.

The striatum-specific context of these findings warrants attention when interpreting the observed KP alterations. Under the neuroinflammatory conditions induced by MPTP, microglial KMO and IDO1 activity is upregulated, shifting the pathway balance towards neurotoxic metabolite accumulation. In this context, the selective elevation of the KA/TRP ratio without a corresponding change in KYN/TRP—observed in the present study—may reflect EMPA-mediated restoration of astrocytic redox state and KAT activity rather than a generalized modulation of TRP catabolism, consistent with the established role of astrocytic redox homeostasis in determining KA biosynthetic capacity.

### EMPA effects on oxidative stress

Our data demonstrate that EMPA administration alone (WT_EMPA group) did not produce significant differences in metabolite levels compared to the WT control group. In contrast, our data show that 3OHK was significantly decreased in the S3KO EMPA-treated group compared to both the S3KO and S3KO MPTP + EMPA-treated groups. This is consistent with published evidence that SIRT3 deletion leads to mitochondrial dysfunction and increased oxidative stress,^49^ creating a metabolic background in which EMPA’s antioxidant effects are more pronounced.

The mechanistic basis for EMPA’s effects on oxidative stress is supported by several independent lines of published evidence. EMPA has been shown to modulate the AMPK–PGC-1α pathway, which is associated with improved mitochondrial integrity, including enhanced mitochondrial biogenesis, mtDNA stability and improved redox homeostasis^50^. Furthermore AMPK activates NRF2 which induces the expression of antioxidant enzymes such as heme-oxygenase-1 (HO-1), NAD(P)H:quinone oxidoreductase-1 (NQO1) and GSH related enzymes (glutathione reductase (GR), glutathione peroxidases (GPXs), and glutathione S-transferases (GSTs)) ^51,52^. Additionally, published studies indicate that EMPA can reduce NF-κB (p65) nuclear translocation in activated microglia, suggesting a potential anti-inflammatory effect^53^. These published mechanisms collectively provide a plausible explanation for the glutathione redox changes observed in our data. The glutathione redox findings in S3KO MPTP + EMPA-treated animals warrant careful mechanistic interpretation. Both GSH and GSSG concentrations were reduced compared to S3KO MPTP controls; however, the disproportionately greater decline in GSSG resulted in a significantly improved GSH/GSSG ratio. This pattern should not be interpreted as simple glutathione pool depletion, but rather as a reflection of reduced glutathione turnover consequent to attenuated oxidative burden. Under the high oxidative load characteristic of the S3KO MPTP condition, both GSH consumption via GPx and GSSG accumulation are elevated, maintaining both forms at high absolute concentrations. EMPA treatment appears to reduce this oxidative drive, leading to decreased GPx activity and/or enhanced GR-mediated GSSG recycling, thereby lowering absolute concentrations of both forms while shifting the redox equilibrium towards a more reduced state. The GSH/GSSG ratio, as the functionally relevant indicator of intracellular redox status, thus reflects genuine improvement in redox homeostasis rather than a paradoxical depletion.

This mechanistic interpretation is independently corroborated by the concurrent reduction of the Oxidative Stress Index in the same experimental group. The convergent directionality of these two biochemically distinct oxidative stress indicators—derived from the glutathione system and the KP, respectively—strengthens the conclusion that EMPA partially restores redox homeostasis in the SIRT3-deficient, MPTP-lesioned background. Reduced levels of the neurotoxic metabolite 3OHK, alongside elevated neuroprotective kynurenines (KA, XA, AA), further indicate a rebalancing of KP flux away from the neurotoxic branch. These processes are likely to improve the redox state of astrocytes, thereby enhancing their capacity for KA production—a mechanism that may contribute to the neuroprotective metabolic profile observed with EMPA treatment in this model. Collectively, these results underscore the value of the S3KO model in dissecting the interplay between mitochondrial dysfunction, oxidative stress, and KP dysregulation in the context of PD.

Altogether, our findings indicate that EMPA shifts kynurenine metabolism towards a neuroprotective metabolic profile by improving redox homeostasis and modulating inflammatory responses, as reflected by the metabolite-level changes observed in the present study. Based on published evidence, the AMPK–NRF2 transcriptional pathway represents the most plausible mechanistic framework contributing to these metabolic alterations; however, direct measurement of these molecular targets was beyond the scope of the present study and requires further validation.

## Limitations of the study

Several limitations of the present study should be acknowledged. First, the designation of observed metabolic changes as consistent with a ‘neuroprotective profile’ is based exclusively on striatal metabolomics data from a pre-clinical rodent model, and refers to established pharmacological properties of KP metabolites—specifically, the capacity of KA, AA, and XA to act as NMDA receptor antagonists and antioxidants as documented in the published literature. In this study design EMPA treatment did not significantly restore striatal DA or DOPAC concentrations in MPTP-treated animals in either genotype, indicating that the observed KP changes occurred independently of dopaminergic recovery. No behavioral assessments, histological analyses, or dopaminergic neuron survival quantifications were performed, and therefore direct functional evidence of neuroprotection cannot be inferred from the present dataset. Future studies incorporating tyrosine hydroxylase (TH) immunohistochemistry, stereological neuron counts, and behavioral endpoints such as rotarod or cylinder testing will be essential to establish whether the metabolic shifts reported here translate to functional neuroprotection in vivo.

Second, the relatively small and unequal group sizes (*n* = 5–10 per group), resulting from technical attrition and, for S3KO groups, from reproductive constraints of the knock-out colony, represent an inherent limitation of the experimental design. Although bootstrap confidence interval analysis confirmed the robustness of the primary KP and glutathione redox findings, the statistical power for detecting smaller effect sizes remains limited, and replication in larger cohorts is warranted.

Third, the mechanistic interpretation of the observed KP alterations—specifically the proposed EMPA-mediated restoration of astrocytic KAT activity—remains indirect, as the present study was conducted at the level of bulk striatal tissue metabolomics without cell-type resolution. As discussed above, the dissociation between unchanged KYN/TRP and selectively elevated KA/TRP ratios is consistent with astrocyte-specific modulation, but this interpretation requires formal validation through single-cell transcriptomics, cell-type-specific enzyme activity assays, and targeted astrocyte/microglia isolation in future studies.

Fourth, the targeted metabolomics panel employed in the present study does not encompass all intermediates of the KP. Quinolinic acid (QA), picolinic acid (PICA), and 3-hydroxyanthranilic acid (3-HANA) were not quantified, representing an inherent constraint of targeted approaches, which are designed around a pre-defined panel optimized for analytical sensitivity and reproducibility within a single run. The absence of QA measurements is a particularly notable limitation, given that QA is a principal neurotoxic endpoint of the KP acting via NMDA receptor agonism, and its inclusion would enable a more complete characterization of neurotoxic branch flux. Furthermore, the enzyme activity estimates used here are based on metabolite concentration ratios (e.g., KA/KYN as a KAT activity proxy) rather than direct enzymatic measurements. Future studies should incorporate QA, PICA, and 3-HANA into the analytical panel, and complement ratio-based proxies with direct IDO, KMO, and KAT isoform activity measurements at the protein or transcriptional level to provide mechanistic validation of the metabolic shifts reported here.

## Conclusion

In conclusion, using a pre-clinical MPTP-induced mouse model of PD, our results demonstrate that EMPA modulates TRP metabolism and the glutathione antioxidant system. EMPA treatment promoted the production of neuroprotective KP metabolites, such as KA, AA and XA, while improving redox balance, particularly in S3KO animals. Notably, these neurometabolic changes occurred independently of dopaminergic recovery, as EMPA did not significantly restore striatal DA or DOPAC levels in MPTP-treated animals, indicating that the observed effects reflect metabolic modulation rather than direct dopaminergic rescue. These findings suggest that EMPA shifts KP metabolism towards a neuroprotective metabolic profile beyond its glucose-lowering action, potentially through the modulation of mitochondrial function and anti-inflammatory pathways, as supported by published evidence. While our study provides metabolic evidence consistent with a neuroprotective potential of SGLT2 inhibition, direct functional validation through TH immunohistochemistry, dopaminergic neuron quantification, and behavioral assessment was not performed and represents an important direction for future research. Further studies are also needed to validate the proposed mechanisms at the proteome and transcriptome levels, and to determine whether similar effects occur in human PD. Overall, our results support the concept that EMPA and related SGLT2 inhibitors could represent promising therapeutic strategies for neurodegenerative diseases through modulation of metabolic and redox pathways.

## Supplementary Information

Supplementary Information 1.

Supplementary Information 2.

Supplementary Information 3.

Supplementary Information 4.

Supplementary Information 5.

Supplementary Information 6.

Supplementary Information 7.

Supplementary Information 8.

## Data Availability

The datasets used and/or analyzed during the current study are available from the corresponding author upon reasonable request.

## Abbreviations

DA: Dopamine
3MT: 3-Methoxytyramine
3OHK: 3-Hydroxykynurenine
AA: Anthranilic acid
AMPK: 5’-Adenosine monophosphate-activated protein kinase
ArAT: Aromatic amino acid aminotransferase
DOPAC: 3,4-Dihydroxyphenylacetic acid
EMPA: Empagliflozin
FDR: False discovery rate
GSH: Glutathione
GSSG: Glutathione disulfide
HVA: Homovanilic acid
I3CA: Indole-3-carboxylic acid
IAA: Indol-3-acetic acid
IDO: Indoleamine 2,3-dioxygenase
ILA: Indole-3-lactic acid
INS: Indoxyl sulfate
IPA: Indole-3-propionic acid
KA: Kynurenic acid
KAT: Kynurenine aminotransferase
KMO: Kynurenine monooxygenase
KP: Kynurenine pathway
KYN: Kynurenine
KYNU: Kynureninase
MDOPA: Methyldopa
MHPGS: 3-Methoxy-4-hydroxyphenylglycol
MPTP: 1-Methyl-4-phenyl-1,2,3,6-tetrahydropyridine
NAD: Nicotinamide adenine dinucleotide
NMDA: *N*-Methyl-*D*-aspartate
NRF2: Nuclear factor erythroid 2-related factor 2
PCA: Principal component analysis
*p*CS: Para-cresyl sulfate
PD: Parkinson’s disease
PGC-α1: Proliferator-activated receptor-γ coactivator
PLP: Pyridoxal 5′-phosphate
PLS-DA: Partial least squares–discriminant analysis
QA: Quinolinic acid
S3KO: Sirtuin3 knock-out
SGLT2: Sodium-glucose cotransporter 2
SN: Substantia nigra
TDO: Tryptophan 2,3-dioxygenase
TMO: Tryptophan 2-monooxygenase
TrD: Tryptophan decarboxylase
TRP: Tryptophan
TYR: Tyrosine
TYRA: Tyramine
UHPLC: Ultra-high performance liquid chromatography
MS/MS: Tandem mass spectrometry
VIP: Variable importance in projection
WT: Wild-type
XA: Xanthurenic acid
