# Supplementary material for: Targeted metabolomics to assess positive effects of empagliflozin in a Parkinson’s disease model: focused on the kynurenine pathway and oxidative stress
Source: Sci Rep. 2026 Jun 4;16:25468. doi: 10.1038/s41598-026-55891-1 (PMC13473509; doi:10.1038/s41598-026-55891-1)
Supplement: Supplementary file 1 [file 41598_2026_55891_MOESM1_ESM.docx]

Volcano plot of the comparison of WT and WT_EMPA groups. The figure shows the fold changes in the quantified metabolites between the groups. FALSE (grey dot): not significantly changed; TRUE (red dot): significantly changed

Volcano plot of the comparison of WT and WT_MPTP groups. The figure shows the fold changes in the quantified metabolites between the groups. FALSE (grey dot): not significantly changed; TRUE (red dot): significantly changed

Volcano plot of the comparison of WT_MPTP and S3KO_MPTP groups. The figure shows the fold changes in the quantified metabolites between the groups. FALSE (grey dot): not significantly changed; TRUE (red dot): significantly changed

Volcano plot of the comparison of S3KO and S3KO_MPTP groups. The figure shows the fold changes in the quantified metabolites between the groups. FALSE (grey dot): not significantly changed; TRUE (red dot): significantly changed

Volcano plot of the comparison of S3KO and S3KO_EMPA groups. The figure shows the fold changes in the quantified metabolites between the groups. FALSE (grey dot): not significantly changed; TRUE (red dot): significantly changed

Volcano plot of the comparison of WT_MPTP and WT_EMPA groups. The figure shows the fold changes in the quantified metabolites between the groups. FALSE (grey dot): not significantly changed; TRUE (red dot): significantly changed

Volcano plot of the comparison of S3KO_EMPA and S3KO_MPTP_EMPA groups. The figure shows the fold changes in the quantified metabolites between the groups. FALSE (grey dot): not significantly changed; TRUE (red dot): significantly changed

Volcano plot of the comparison of S3KO_MPTP and S3KO_EMPA groups. The figure shows the fold changes in the quantified metabolites between the groups. FALSE (grey dot): not significantly changed; TRUE (red dot): significantly changed

Volcano plot of the comparison of WT and S3KO groups. The figure shows the fold changes in the quantified metabolites between the groups. FALSE (grey dot): not significantly changed; TRUE (red dot): significantly changed
