# Supplementary material for: Targeted metabolomics to assess positive effects of empagliflozin in a Parkinson’s disease model: focused on the kynurenine pathway and oxidative stress
Source: Sci Rep. 2026 Jun 4;16:25468. doi: 10.1038/s41598-026-55891-1 (PMC13473509; doi:10.1038/s41598-026-55891-1)
Supplement: Supplementary file 2 [file 41598_2026_55891_MOESM2_ESM.docx]

Boxplots of the comparison in DA levels between the 8 group.

Boxplots of the comparison in INS/TRP_TNA levels between the 8 group.

Boxplots of the comparison in IPA levels between the 8 group.

Boxplots of the comparison in KYN levels between the 8 group.

Boxplots of the comparison in INS levels between the 8 group.

Boxplots of the comparison in ILA levels between the 8 group.

Boxplots of the comparison in IAA/TRP_TMO_TrD_ArAT levels between the 8 group.

Boxplots of the comparison in IAA levels between the 8 group.

Boxplots of the comparison in I3CA levels between the 8 group.

Boxplots of the comparison in KYN/TRP_IDOs_TDO levels between the 8 group.

Boxplots of the comparison in HVA levels between the 8 group.

Boxplots of the comparison in MHPGS levels between the 8 group.

Boxplots of the comparison in MDOPA levels between the 8 group.

Boxplots of the comparison in NAD levels between the 8 group.

Boxplots of the comparison in 3MT levels between the 8 group.

Boxplots of the comparison in TYRA levels between the 8 group.

Boxplots of the comparison in TYR levels between the 8 group.

Boxplots of the comparison in TRP levels between the 8 group.

Boxplots of the comparison in PLP levels between the 8 group.

Boxplots of the comparison in pCS/TYR levels between the 8 group.

Boxplots of the comparison in pCS levels between the 8 group.

Boxplots of the comparison in DOPAC levels between the 8 group.
