# Supplementary material for: Targeted metabolomics to assess positive effects of empagliflozin in a Parkinson’s disease model: focused on the kynurenine pathway and oxidative stress
Source: Sci Rep. 2026 Jun 4;16:25468. doi: 10.1038/s41598-026-55891-1 (PMC13473509; doi:10.1038/s41598-026-55891-1)
Supplement: Supplementary file 3 [file 41598_2026_55891_MOESM3_ESM.docx]

| **Analyte** | **Precursor ion (m/z)** | **Product ion (m/z)** | **DP (V)** | **CE (V)** | **RT (min)** | **Polarity** |
| --- | --- | --- | --- | --- | --- | --- |
| TRP | 205.1 | 188.1 | 39 | 32 | 8.00 | + |
| KYN | 209.1 | 146.1 | 40 | 23 | 4.64 | + |
| KA | 190.1 | 144.1 | 20 | 25 | 9.50 | + |
| AA | 138.05 | 120.05 | 44 | 15 | 10.60 | + |
| XA | 206.1 | 160.1 | 30 | 25 | 9.11 | + |
| 3OHK | 225.1 | 162.1 | 20 | 27 | 2.50 | + |
| NAD | 664.2 | 136.1 | 85 | 50 | 2.00 | + |
| PLP | 248.1 | 150.1 | 50 | 22 | 1.90 | + |
| IAA | 176.1 | 130.1 | 55 | 20 | 12.68 | + |
| ILA | 206.1 | 188.1 | 50 | 13 | 12.00 | + |
| IPA | 190.1 | 130.1 | 50 | 19 | 13.00 | + |
| I3CA | 146.1 | 118.1 | 50 | 19 | 12.40 | + |
| INS | 211.9 | 131.9 | −50 | −25 | 11.80 | − |
| pCS | 186.9 | 107.0 | −50 | −26 | 12.70 | − |
| DA | 154.1 | 137.1 | 55 | 13 | 1.86 | + |
| DOPAC | 167.0 | 122.9 | −40 | −11 | 8.28 | − |
| HVA | 181.0 | 122.0 | −70 | −18 | 10.50 | − |
| 3MT | 168.2 | 119.0 | 35 | 24 | 3.72 | + |
| TYR | 182.1 | 136.1 | 110 | 42 | 2.80 | + |
| TYRA | 138.1 | 121.1 | 45 | 14 | 2.54 | + |
| MDOPA | 212.1 | 149.1 | 130 | 19 | 4.23 | + |
| MHPGS | 263.0 | 165.0 | −19 | −27 | 3.00 | − |
| GSH | 308.2 | 179.1 | 50 | 14 | 1.76 | + |
| GSSG | 614.4 | 485.1 | 50 | 17 | 2.58 | + |

MRM transitions, ion source parameters, and chromatographic retention times for all quantified analytes. DP = declustering potential; CE = collision energy; RT = retention time. Polarity: (+) positive ESI mode, (−) negative ESI mode.

Abbreviation

| DA | dopamine |
| --- | --- |
| 3MT | 3-Methoxytyramine |
| 3OHK | 3-hydroxykynurenine |
| AA | anthranilic acid |
| DOPAC | 3,4-Dihydroxyphenylacetic acid |
|  |  |
| GSH | glutathione |
| GSSG | glutathione disulfide |
| HVA | homovanilic acid |
| I3CA | Indole-3-carboxylic acid |
| IAA | indol-3-acetic acid |
| IDO | indoleamine 2,3-dioxygenase |
| ILA | indole-3-lactic acid |
| INS | indoxyl sulfate |
| IPA | Indole-3-propionic acid |
| KA | kynurenic acid |
|  |  |
|  |  |
| KYN | kynurenine |
|  |  |
| MDOPA | methyldopa |
| MHPGS | 3-methoxy-4-hydroxyphenylglycol |
|  |  |
| NAD | Nicotinamide adenine dinucleotide |
| *p*CS | para-cresyl sulfate |
|  |  |
| PLP | pyridoxal 5′-phosphate |
| PLS-DA | partial least squares–discriminant analysis |
|  |  |
|  |  |
|  |  |
| TRP | tryptophan |
| TYR | tyrosine |
| TYRA | tyramine |
|  |  |
| XA | xanthurenic acid |
