# Supplementary material for: Targeted metabolomics to assess positive effects of empagliflozin in a Parkinson’s disease model: focused on the kynurenine pathway and oxidative stress
Source: Sci Rep. 2026 Jun 4;16:25468. doi: 10.1038/s41598-026-55891-1 (PMC13473509; doi:10.1038/s41598-026-55891-1)
Supplement: Supplementary file 4 [file 41598_2026_55891_MOESM5_ESM.docx]

**Effect sizes and bootstrap 95% confidence intervals for primary endpoints**

| **Metabolite** | **Comparison** | **n (ref)** | **n (comp)** | **Mean ref** | **Mean comp** | **Cohen's d** | **95% CI (bootstrap)** |
| --- | --- | --- | --- | --- | --- | --- | --- |
| ***A) Kynurenine pathway metabolites*** | | | | | | | |
| KA | WT_MPTP_EMPA vs WT | 9 | 9 | 0.43 | 3.94 | **−3.19** | **[2.42, 8.35]** |
| KA | WT_MPTP_EMPA vs WT_MPTP | 7 | 9 | 0.74 | 3.94 | **−2.70** | **[2.06, 6.57]** |
| KA | S3KO_MPTP_EMPA vs S3KO | 5 | 8 | 0.45 | 3.55 | **−3.16** | **[2.43, 7.11]** |
| KA | S3KO_MPTP_EMPA vs S3KO_MPTP | 8 | 8 | 1.10 | 3.55 | **−2.31** | **[1.54, 4.23]** |
| AA | WT_MPTP_EMPA vs WT | 9 | 9 | 0.17 | 0.60 | **−2.14** | **[1.36, 4.53]** |
| AA | WT_MPTP_EMPA vs WT_MPTP | 8 | 9 | 0.22 | 0.60 | **−1.76** | **[1.00, 3.62]** |
| AA | S3KO_MPTP_EMPA vs S3KO | 5 | 8 | 0.23 | 0.79 | **−1.57** | **[0.74, 3.98]** |
| AA | S3KO_MPTP_EMPA vs S3KO_MPTP | 9 | 8 | 0.25 | 0.79 | **−1.72** | **[0.80, 4.21]** |
| XA | WT_MPTP_EMPA vs WT | 9 | 8 | 2.14 | 11.41 | **−1.32** | **[0.64, 2.93]** |
| XA | WT_MPTP_EMPA vs WT_MPTP | 8 | 8 | 4.12 | 11.41 | **−1.00** | **[0.17, 2.21]** |
| XA | S3KO_MPTP_EMPA vs S3KO | 5 | 8 | 0.46 | 8.40 | **−2.04** | **[1.25, 7.55]** |
| XA | S3KO_MPTP_EMPA vs S3KO_MPTP | 9 | 8 | 1.93 | 8.40 | **−1.84** | **[0.92, 5.21]** |
| KA/TRP | WT_MPTP_EMPA vs WT | 9 | 8 | 0.000 | 0.0001 | **−2.16** | **[1.60, 5.41]** |
| KA/TRP | WT_MPTP_EMPA vs WT_MPTP | 7 | 8 | 0.000 | 0.0001 | **−1.75** | **[1.25, 3.82]** |
| KA/TRP | S3KO_MPTP_EMPA vs S3KO | 5 | 7 | 0.000 | 0.0001 | **−3.66** | **[2.80, 7.94]** |
| KA/TRP | S3KO_MPTP_EMPA vs S3KO_MPTP | 8 | 7 | 0.000 | 0.0001 | **−1.64** | **[0.66, 3.91]** |
| OSI | WT_MPTP_EMPA vs WT | 9 | 9 | 19.97 | 2.20 | **0.91** | **[−6.74, −0.72]** |
| OSI | WT_MPTP_EMPA vs WT_MPTP | 8 | 9 | 6.02 | 2.20 | **1.45** | **[−3.13, −0.81]** |
| OSI | S3KO_MPTP_EMPA vs S3KO | 5 | 7 | 25.39 | 2.05 | **3.46** | **[−17.09, −2.47]** |
| OSI | S3KO_MPTP_EMPA vs S3KO_MPTP | 8 | 7 | 8.29 | 2.05 | **1.81** | **[−3.80, −1.23]** |
| ***B) Glutathione redox parameters*** | | | | | | | |
| GSH | S3KO_MPTP_EMPA vs S3KO | 5 | 8 | 148800 | 102463 | **1.18** | **[−3.12, −0.17]** |
| GSH | S3KO_MPTP_EMPA vs S3KO_MPTP | 9 | 8 | 174533 | 102463 | **1.72** | **[−3.75, −0.76]** |
| GSSG | S3KO_MPTP_EMPA vs S3KO | 5 | 8 | 54580 | 13661 | **1.32** | **[−6.00, −0.33]** |
| GSSG | S3KO_MPTP_EMPA vs S3KO_MPTP | 9 | 8 | 52167 | 13661 | **2.78** | **[−6.13, −1.78]** |
| GSH/GSSG | S3KO_MPTP_EMPA vs S3KO | 5 | 8 | 5.79 | 7.96 | −0.61 | [−1.31, 4.93] |
| GSH/GSSG | S3KO_MPTP_EMPA vs S3KO_MPTP | 9 | 8 | 3.90 | 7.96 | **−1.95** | **[0.83, 5.30]** |
| ***C) Dopaminergic markers (MPTP lesion validation)*** | | | | | | | |
| DA | WT_MPTP_EMPA vs WT | 9 | 9 | 38100 | 8451 | **4.03** | **[−6.94, −3.16]** |
| DA | S3KO_MPTP_EMPA vs S3KO | 5 | 8 | 34540 | 8939 | **6.50** | **[−17.37, −5.01]** |
| DOPAC | WT_MPTP_EMPA vs WT | 10 | 9 | 14057 | 5232 | **2.40** | **[−4.19, −1.78]** |
| DOPAC | S3KO_MPTP_EMPA vs S3KO | 5 | 8 | 11718 | 4833 | **2.61** | **[−6.27, −1.90]** |

OSI = Oxidative Stress Index (3-OHK/(KA+AA+XA)); KA = kynurenic acid; AA = anthranilic acid; XA = xanthurenic acid; DA = dopamine; DOPAC = 3,4-dihydroxyphenylacetic acid.

Cohen's d: effect size calculated using pooled SD. Interpretation thresholds: negligible |d| < 0.2; small 0.2–0.5; medium 0.5–0.8; large ≥ 0.8.

95% CI: bootstrap percentile confidence interval (n = 10,000 iterations, seed = 42).

Bold red values: 95% CI excludes zero, indicating a robust effect independent of sample size.

p_FDR: Benjamini–Hochberg FDR-adjusted p-value. Mean values reported in original measurement units.

Comp = comparison group; Ref = reference group. n = number of animals per group after exclusion of non-finite values.
