# Supplementary material for: Targeted metabolomics to assess positive effects of empagliflozin in a Parkinson’s disease model: focused on the kynurenine pathway and oxidative stress
Source: Sci Rep. 2026 Jun 4;16:25468. doi: 10.1038/s41598-026-55891-1 (PMC13473509; doi:10.1038/s41598-026-55891-1)
Supplement: Supplementary file 5 [file 41598_2026_55891_MOESM6_ESM.docx]

| Metabolite | VIP score |
| --- | --- |
| KA, | 2.03369185030443 |
| DA, | 1.71231520280336 |
| KA/KYN_KATs, | 1.70883433396859 |
| KA/TRP, | 1.64847657998961 |
| AA, | 1.53155694070737 |
| DOPAC, | 1.51765202020786 |
| AA/KYN_KYNU, | 1.38810030295185 |
| XA, | 1.36485079994225 |
| 3MT, | 1.30976894878749 |
| XA/3OHK_KATIII, | 1.16384094026063 |
| TYRA, | 1.15105546979953 |
| INS, | 1.14372116358928 |
| IAA/TRP_TMO_TrD_ArAT, | 1.12970994016859 |
| Oxidative_Stress_Index = 3OHK/(KA+AA+XA), | 1.03543130648009 |
| INS/TRP_TNA, | 0.900053736106831 |
| GSH, | 0.843059311178453 |
| GSSG, | 0.83146761340343 |
| TRP, | 0.711457527881081 |
| IAA, | 0.701509989427653 |
| HVA, | 0.668682140896395 |
| MHPGS, | 0.617640883731759 |
| TYR, | 0.574950622723984 |
| NAD, | 0.5582662062356 |
| GSH/GSSG, | 0.530993123612661 |
| 3OHK, | 0.523410780577297 |
| KYN, | 0.474611233350073 |
| pCS, | 0.30902368299839 |
| ILA, | 0.28944011720042 |
| pCS/TYR, | 0.284889545224321 |
| KYN/TRP_IDOs_TDO, | 0.202078915389309 |
| PLP, | 0.122976096243943 |
| I3CA, | 0.116509707883265 |
| 3OHK/KYN_KMO, | 0.0991776497377376 |
| MDOPA, | 0.0438006749419945 |
| IPA, | 0.0171038292847816 |

**Variable Importance in Projection (VIP) scores of the quantified metabolites**

Abbreviations

| DA | dopamine |
| --- | --- |
| 3MT | 3-Methoxytyramine |
| 3OHK | 3-hydroxykynurenine |
| AA | anthranilic acid |
| AMPK | 5’-adenosine monophosphate-activated protein kinase |
| ArAT | Aromatic amino acid aminotransferase |
| DOPAC | 3,4-Dihydroxyphenylacetic acid |
| GSH | glutathione |
| GSSG | glutathione disulfide |
| HVA | homovanilic acid |
| I3CA | Indole-3-carboxylic acid |
| IAA | indol-3-acetic acid |
| IDO | indoleamine 2,3-dioxygenase |
| ILA | indole-3-lactic acid |
| INS | indoxyl sulfate |
| IPA | Indole-3-propionic acid |
| KA | kynurenic acid |
| KAT | kynurenine aminotransferase |
| KMO | kynurenine monooxygenase |
| KYN | kynurenine |
| KYNU | kynureninase |
| MDOPA | methyldopa |
| MHPGS | 3-methoxy-4-hydroxyphenylglycol |
| MPTP | 1-methyl-4-phenyl-1,2,3,6-tetrahydropyridine |
| NAD | Nicotinamide adenine dinucleotide |
| *p*CS | para-cresyl sulfate |
| PD | Parkinson's disease |
| PLP | pyridoxal 5′-phosphate |
| PLS-DA | partial least squares–discriminant analysis |
| S3KO | Sirtuin3 knock-out |
| SGLT2 | Sodium-glucose cotransporter 2 |
| TDO | Tryptophan 2,3-dioxygenase |
| TMO | tryptophan 2-monooxygenase |
| TrD | Tryptophan decarboxylase |
| TRP | tryptophan |
| TYR | tyrosine |
| TYRA | tyramine |
| VIP | Variable Importance in Projection |
| XA | xanthurenic acid |
