# Supplementary material for: Targeted metabolomics to assess positive effects of empagliflozin in a Parkinson’s disease model: focused on the kynurenine pathway and oxidative stress
Source: Sci Rep. 2026 Jun 4;16:25468. doi: 10.1038/s41598-026-55891-1 (PMC13473509; doi:10.1038/s41598-026-55891-1)
Supplement: Supplementary file 6 [file 41598_2026_55891_MOESM8_ESM.docx]

**Results of the univariate statistical analysis**.

The table shows the comparisons of the levels of all metabolites between the groups (separately within the WT and S3KO). Welch’s ANOVA, Games-Howell post-hoc; test; *: p < 0.05; **: p < 0,01; ***: p < 0.001

| Metabolite | Group type | Group1 | Group2 | GH post hoc *p* | Welch_ANOVA *p* | Sign. level |
| --- | --- | --- | --- | --- | --- | --- |
| TYR | WT | WT_MPTP | WT | 0,693274 | 0,008468 |  |
| TYR | WT | WT_EMPA | WT | 0,077204 | 0,008468 |  |
| TYR | WT | WT_MPTP_EMPA | WT | 0,231489 | 0,008468 |  |
| TYR | WT | WT_MPTP | WT_MPTP | | 0,008468 |  |
| TYR | WT | WT_EMPA | WT_MPTP | 0,042333 | 0,008468 | * |
| TYR | WT | WT_MPTP_EMPA | WT_MPTP | 0,111672 | 0,008468 |  |
| TYR | WT | WT_MPTP | WT_EMPA | | 0,008468 |  |
| TYR | WT | WT_EMPA | WT_EMPA | | 0,008468 |  |
| TYR | WT | WT_MPTP_EMPA | WT_EMPA | 0,89468 | 0,008468 |  |
| TYR | S3KO | S3KO_MPTP | S3KO | 0,027038 | 0,00167 | * |
| TYR | S3KO | S3KO_EMPA | S3KO | 0,029164 | 0,00167 | * |
| TYR | S3KO | S3KO_MPTP_EMPA | S3KO | 0,002592 | 0,00167 | ** |
| TYR | S3KO | S3KO_MPTP | S3KO_MPTP | | 0,00167 |  |
| TYR | S3KO | S3KO_EMPA | S3KO_MPTP | 0,997729 | 0,00167 |  |
| TYR | S3KO | S3KO_MPTP_EMPA | S3KO_MPTP | 0,511099 | 0,00167 |  |
| TYR | S3KO | S3KO_MPTP | S3KO_EMPA | | 0,00167 |  |
| TYR | S3KO | S3KO_EMPA | S3KO_EMPA | | 0,00167 |  |
| TYR | S3KO | S3KO_MPTP_EMPA | S3KO_EMPA | 0,626078 | 0,00167 |  |
| MDOPA | WT | WT_MPTP | WT | 0,323902 | 0,014449 |  |
| MDOPA | WT | WT_EMPA | WT | 0,279202 | 0,014449 |  |
| MDOPA | WT | WT_MPTP_EMPA | WT | 0,911959 | 0,014449 |  |
| MDOPA | WT | WT_MPTP | WT_MPTP | | 0,014449 |  |
| MDOPA | WT | WT_EMPA | WT_MPTP | 0,017377 | 0,014449 | * |
| MDOPA | WT | WT_MPTP_EMPA | WT_MPTP | 0,1717 | 0,014449 |  |
| MDOPA | WT | WT_MPTP | WT_EMPA | | 0,014449 |  |
| MDOPA | WT | WT_EMPA | WT_EMPA | | 0,014449 |  |
| MDOPA | WT | WT_MPTP_EMPA | WT_EMPA | 0,705895 | 0,014449 |  |
| MDOPA | S3KO | S3KO_MPTP | S3KO | 0,996268 | 0,908619 |  |
| MDOPA | S3KO | S3KO_EMPA | S3KO | 0,981762 | 0,908619 |  |
| MDOPA | S3KO | S3KO_MPTP_EMPA | S3KO | 0,946162 | 0,908619 |  |
| MDOPA | S3KO | S3KO_MPTP | S3KO_MPTP | | 0,908619 |  |
| MDOPA | S3KO | S3KO_EMPA | S3KO_MPTP | 0,965645 | 0,908619 |  |
| MDOPA | S3KO | S3KO_MPTP_EMPA | S3KO_MPTP | 0,995276 | 0,908619 |  |
| MDOPA | S3KO | S3KO_MPTP | S3KO_EMPA | | 0,908619 |  |
| MDOPA | S3KO | S3KO_EMPA | S3KO_EMPA | | 0,908619 |  |
| MDOPA | S3KO | S3KO_MPTP_EMPA | S3KO_EMPA | 0,881235 | 0,908619 |  |
| DA | WT | WT_MPTP | WT | 7,3E-06 | 5,75E-08 | *** |
| DA | WT | WT_EMPA | WT | 0,836989 | 5,75E-08 |  |
| DA | WT | WT_MPTP_EMPA | WT | 3,79E-06 | 5,75E-08 | *** |
| DA | WT | WT_MPTP | WT_MPTP | | 5,75E-08 |  |
| DA | WT | WT_EMPA | WT_MPTP | 3,76E-05 | 5,75E-08 | *** |
| DA | WT | WT_MPTP_EMPA | WT_MPTP | 0,997194 | 5,75E-08 |  |
| DA | WT | WT_MPTP | WT_EMPA | | 5,75E-08 |  |
| DA | WT | WT_EMPA | WT_EMPA | | 5,75E-08 |  |
| DA | WT | WT_MPTP_EMPA | WT_EMPA | 2,06E-05 | 5,75E-08 | *** |
| DA | S3KO | S3KO_MPTP | S3KO | 0,000543 | 6,83E-06 | *** |
| DA | S3KO | S3KO_EMPA | S3KO | 0,660465 | 6,83E-06 |  |
| DA | S3KO | S3KO_MPTP_EMPA | S3KO | 0,000888 | 6,83E-06 | *** |
| DA | S3KO | S3KO_MPTP | S3KO_MPTP | | 6,83E-06 |  |
| DA | S3KO | S3KO_EMPA | S3KO_MPTP | 0,005032 | 6,83E-06 | ** |
| DA | S3KO | S3KO_MPTP_EMPA | S3KO_MPTP | 0,801103 | 6,83E-06 |  |
| DA | S3KO | S3KO_MPTP | S3KO_EMPA | | 6,83E-06 |  |
| DA | S3KO | S3KO_EMPA | S3KO_EMPA | | 6,83E-06 |  |
| DA | S3KO | S3KO_MPTP_EMPA | S3KO_EMPA | 0,006167 | 6,83E-06 | ** |
| 3MT | WT | WT_MPTP | WT | 3,79E-05 | 2,11E-05 | *** |
| 3MT | WT | WT_EMPA | WT | 0,067411 | 2,11E-05 |  |
| 3MT | WT | WT_MPTP_EMPA | WT | 0,00014 | 2,11E-05 | *** |
| 3MT | WT | WT_MPTP | WT_MPTP | | 2,11E-05 |  |
| 3MT | WT | WT_EMPA | WT_MPTP | 0,003558 | 2,11E-05 | ** |
| 3MT | WT | WT_MPTP_EMPA | WT_MPTP | 0,865856 | 2,11E-05 |  |
| 3MT | WT | WT_MPTP | WT_EMPA | | 2,11E-05 |  |
| 3MT | WT | WT_EMPA | WT_EMPA | | 2,11E-05 |  |
| 3MT | WT | WT_MPTP_EMPA | WT_EMPA | 0,017479 | 2,11E-05 | * |
| 3MT | S3KO | S3KO_MPTP | S3KO | 0,023104 | 0,015564 | * |
| 3MT | S3KO | S3KO_EMPA | S3KO | 0,728799 | 0,015564 |  |
| 3MT | S3KO | S3KO_MPTP_EMPA | S3KO | 0,239105 | 0,015564 |  |
| 3MT | S3KO | S3KO_MPTP | S3KO_MPTP | | 0,015564 |  |
| 3MT | S3KO | S3KO_EMPA | S3KO_MPTP | 0,052774 | 0,015564 |  |
| 3MT | S3KO | S3KO_MPTP_EMPA | S3KO_MPTP | 0,682693 | 0,015564 |  |
| 3MT | S3KO | S3KO_MPTP | S3KO_EMPA | | 0,015564 |  |
| 3MT | S3KO | S3KO_EMPA | S3KO_EMPA | | 0,015564 |  |
| 3MT | S3KO | S3KO_MPTP_EMPA | S3KO_EMPA | 0,173073 | 0,015564 |  |
| DOPAC | WT | WT_MPTP | WT | 0,000248 | 1,7E-05 | *** |
| DOPAC | WT | WT_EMPA | WT | 0,164613 | 1,7E-05 |  |
| DOPAC | WT | WT_MPTP_EMPA | WT | 0,00058 | 1,7E-05 | *** |
| DOPAC | WT | WT_MPTP | WT_MPTP | | 1,7E-05 |  |
| DOPAC | WT | WT_EMPA | WT_MPTP | 0,006993 | 1,7E-05 | ** |
| DOPAC | WT | WT_MPTP_EMPA | WT_MPTP | 0,402852 | 1,7E-05 |  |
| DOPAC | WT | WT_MPTP | WT_EMPA | | 1,7E-05 |  |
| DOPAC | WT | WT_EMPA | WT_EMPA | | 1,7E-05 |  |
| DOPAC | WT | WT_MPTP_EMPA | WT_EMPA | 0,032355 | 1,7E-05 | * |
| DOPAC | S3KO | S3KO_MPTP | S3KO | 0,028084 | 0,000826 | * |
| DOPAC | S3KO | S3KO_EMPA | S3KO | 0,999909 | 0,000826 |  |
| DOPAC | S3KO | S3KO_MPTP_EMPA | S3KO | 0,044784 | 0,000826 | * |
| DOPAC | S3KO | S3KO_MPTP | S3KO_MPTP | | 0,000826 |  |
| DOPAC | S3KO | S3KO_EMPA | S3KO_MPTP | 0,009222 | 0,000826 | ** |
| DOPAC | S3KO | S3KO_MPTP_EMPA | S3KO_MPTP | 0,429607 | 0,000826 |  |
| DOPAC | S3KO | S3KO_MPTP | S3KO_EMPA | | 0,000826 |  |
| DOPAC | S3KO | S3KO_EMPA | S3KO_EMPA | | 0,000826 |  |
| DOPAC | S3KO | S3KO_MPTP_EMPA | S3KO_EMPA | 0,016381 | 0,000826 | * |
| HVA | WT | WT_MPTP | WT | 0,023391 | 0,024245 | * |
| HVA | WT | WT_EMPA | WT | 0,999656 | 0,024245 |  |
| HVA | WT | WT_MPTP_EMPA | WT | 0,415402 | 0,024245 |  |
| HVA | WT | WT_MPTP | WT_MPTP | | 0,024245 |  |
| HVA | WT | WT_EMPA | WT_MPTP | 0,080897 | 0,024245 |  |
| HVA | WT | WT_MPTP_EMPA | WT_MPTP | 0,603801 | 0,024245 |  |
| HVA | WT | WT_MPTP | WT_EMPA | | 0,024245 |  |
| HVA | WT | WT_EMPA | WT_EMPA | | 0,024245 |  |
| HVA | WT | WT_MPTP_EMPA | WT_EMPA | 0,519957 | 0,024245 |  |
| HVA | S3KO | S3KO_MPTP | S3KO | 0,102374 | 0,021209 |  |
| HVA | S3KO | S3KO_EMPA | S3KO | 0,999991 | 0,021209 |  |
| HVA | S3KO | S3KO_MPTP_EMPA | S3KO | 0,508315 | 0,021209 |  |
| HVA | S3KO | S3KO_MPTP | S3KO_MPTP | | 0,021209 |  |
| HVA | S3KO | S3KO_EMPA | S3KO_MPTP | 0,039762 | 0,021209 | * |
| HVA | S3KO | S3KO_MPTP_EMPA | S3KO_MPTP | 0,416827 | 0,021209 |  |
| HVA | S3KO | S3KO_MPTP | S3KO_EMPA | | 0,021209 |  |
| HVA | S3KO | S3KO_EMPA | S3KO_EMPA | | 0,021209 |  |
| HVA | S3KO | S3KO_MPTP_EMPA | S3KO_EMPA | 0,417972 | 0,021209 |  |
| MHPGS | WT | WT_MPTP | WT | 0,436328 | 0,139006 |  |
| MHPGS | WT | WT_EMPA | WT | 0,990866 | 0,139006 |  |
| MHPGS | WT | WT_MPTP_EMPA | WT | 0,272441 | 0,139006 |  |
| MHPGS | WT | WT_MPTP | WT_MPTP | | 0,139006 |  |
| MHPGS | WT | WT_EMPA | WT_MPTP | 0,632514 | 0,139006 |  |
| MHPGS | WT | WT_MPTP_EMPA | WT_MPTP | 0,077158 | 0,139006 |  |
| MHPGS | WT | WT_MPTP | WT_EMPA | | 0,139006 |  |
| MHPGS | WT | WT_EMPA | WT_EMPA | | 0,139006 |  |
| MHPGS | WT | WT_MPTP_EMPA | WT_EMPA | 0,793726 | 0,139006 |  |
| MHPGS | S3KO | S3KO_MPTP | S3KO | 0,953702 | 0,452664 |  |
| MHPGS | S3KO | S3KO_EMPA | S3KO | 0,942695 | 0,452664 |  |
| MHPGS | S3KO | S3KO_MPTP_EMPA | S3KO | 0,711118 | 0,452664 |  |
| MHPGS | S3KO | S3KO_MPTP | S3KO_MPTP | | 0,452664 |  |
| MHPGS | S3KO | S3KO_EMPA | S3KO_MPTP | 0,7058 | 0,452664 |  |
| MHPGS | S3KO | S3KO_MPTP_EMPA | S3KO_MPTP | 0,426563 | 0,452664 |  |
| MHPGS | S3KO | S3KO_MPTP | S3KO_EMPA | | 0,452664 |  |
| MHPGS | S3KO | S3KO_EMPA | S3KO_EMPA | | 0,452664 |  |
| MHPGS | S3KO | S3KO_MPTP_EMPA | S3KO_EMPA | 0,947358 | 0,452664 |  |
| TYRA | WT | WT_MPTP | WT | 5,84E-05 | 3,65E-06 | *** |
| TYRA | WT | WT_EMPA | WT | 0,474163 | 3,65E-06 |  |
| TYRA | WT | WT_MPTP_EMPA | WT | 0,000117 | 3,65E-06 | *** |
| TYRA | WT | WT_MPTP | WT_MPTP | | 3,65E-06 |  |
| TYRA | WT | WT_EMPA | WT_MPTP | 0,01005 | 3,65E-06 | * |
| TYRA | WT | WT_MPTP_EMPA | WT_MPTP | 0,256135 | 3,65E-06 |  |
| TYRA | WT | WT_MPTP | WT_EMPA | | 3,65E-06 |  |
| TYRA | WT | WT_EMPA | WT_EMPA | | 3,65E-06 |  |
| TYRA | WT | WT_MPTP_EMPA | WT_EMPA | 0,016171 | 3,65E-06 | * |
| TYRA | S3KO | S3KO_MPTP | S3KO | 0,371256 | 0,038092 |  |
| TYRA | S3KO | S3KO_EMPA | S3KO | 0,23288 | 0,038092 |  |
| TYRA | S3KO | S3KO_MPTP_EMPA | S3KO | 0,440536 | 0,038092 |  |
| TYRA | S3KO | S3KO_MPTP | S3KO_MPTP | | 0,038092 |  |
| TYRA | S3KO | S3KO_EMPA | S3KO_MPTP | 0,06286 | 0,038092 |  |
| TYRA | S3KO | S3KO_MPTP_EMPA | S3KO_MPTP | 0,993077 | 0,038092 |  |
| TYRA | S3KO | S3KO_MPTP | S3KO_EMPA | | 0,038092 |  |
| TYRA | S3KO | S3KO_EMPA | S3KO_EMPA | | 0,038092 |  |
| TYRA | S3KO | S3KO_MPTP_EMPA | S3KO_EMPA | 0,068904 | 0,038092 |  |
| TRP | WT | WT_MPTP | WT | 0,675654 | 0,040485 |  |
| TRP | WT | WT_EMPA | WT | 0,18612 | 0,040485 |  |
| TRP | WT | WT_MPTP_EMPA | WT | 0,441463 | 0,040485 |  |
| TRP | WT | WT_MPTP | WT_MPTP | | 0,040485 |  |
| TRP | WT | WT_EMPA | WT_MPTP | 0,069325 | 0,040485 |  |
| TRP | WT | WT_MPTP_EMPA | WT_MPTP | 0,225023 | 0,040485 |  |
| TRP | WT | WT_MPTP | WT_EMPA | | 0,040485 |  |
| TRP | WT | WT_EMPA | WT_EMPA | | 0,040485 |  |
| TRP | WT | WT_MPTP_EMPA | WT_EMPA | 0,996723 | 0,040485 |  |
| TRP | S3KO | S3KO_MPTP | S3KO | 0,931996 | 0,225483 |  |
| TRP | S3KO | S3KO_EMPA | S3KO | 0,979155 | 0,225483 |  |
| TRP | S3KO | S3KO_MPTP_EMPA | S3KO | 0,506776 | 0,225483 |  |
| TRP | S3KO | S3KO_MPTP | S3KO_MPTP | | 0,225483 |  |
| TRP | S3KO | S3KO_EMPA | S3KO_MPTP | 0,999631 | 0,225483 |  |
| TRP | S3KO | S3KO_MPTP_EMPA | S3KO_MPTP | 0,148598 | 0,225483 |  |
| TRP | S3KO | S3KO_MPTP | S3KO_EMPA | | 0,225483 |  |
| TRP | S3KO | S3KO_EMPA | S3KO_EMPA | | 0,225483 |  |
| TRP | S3KO | S3KO_MPTP_EMPA | S3KO_EMPA | 0,324381 | 0,225483 |  |
| KYN | WT | WT_MPTP | WT | 0,999014 | 0,135149 |  |
| KYN | WT | WT_EMPA | WT | 0,202453 | 0,135149 |  |
| KYN | WT | WT_MPTP_EMPA | WT | 0,395175 | 0,135149 |  |
| KYN | WT | WT_MPTP | WT_MPTP | | 0,135149 |  |
| KYN | WT | WT_EMPA | WT_MPTP | 0,254563 | 0,135149 |  |
| KYN | WT | WT_MPTP_EMPA | WT_MPTP | 0,516396 | 0,135149 |  |
| KYN | WT | WT_MPTP | WT_EMPA | | 0,135149 |  |
| KYN | WT | WT_EMPA | WT_EMPA | | 0,135149 |  |
| KYN | WT | WT_MPTP_EMPA | WT_EMPA | 0,880253 | 0,135149 |  |
| KYN | S3KO | S3KO_MPTP | S3KO | 0,600628 | 0,164059 |  |
| KYN | S3KO | S3KO_EMPA | S3KO | 0,674539 | 0,164059 |  |
| KYN | S3KO | S3KO_MPTP_EMPA | S3KO | 0,489066 | 0,164059 |  |
| KYN | S3KO | S3KO_MPTP | S3KO_MPTP | | 0,164059 |  |
| KYN | S3KO | S3KO_EMPA | S3KO_MPTP | 0,999955 | 0,164059 |  |
| KYN | S3KO | S3KO_MPTP_EMPA | S3KO_MPTP | 0,151526 | 0,164059 |  |
| KYN | S3KO | S3KO_MPTP | S3KO_EMPA | | 0,164059 |  |
| KYN | S3KO | S3KO_EMPA | S3KO_EMPA | | 0,164059 |  |
| KYN | S3KO | S3KO_MPTP_EMPA | S3KO_EMPA | 0,188985 | 0,164059 |  |
| KA | WT | WT_MPTP | WT | 0,093408 | 0,000116 |  |
| KA | WT | WT_EMPA | WT | 0,343916 | 0,000116 |  |
| KA | WT | WT_MPTP_EMPA | WT | 0,00059 | 0,000116 | *** |
| KA | WT | WT_MPTP | WT_MPTP | | 0,000116 |  |
| KA | WT | WT_EMPA | WT_MPTP | 0,9959 | 0,000116 |  |
| KA | WT | WT_MPTP_EMPA | WT_MPTP | 0,001008 | 0,000116 | ** |
| KA | WT | WT_MPTP | WT_EMPA | | 0,000116 |  |
| KA | WT | WT_EMPA | WT_EMPA | | 0,000116 |  |
| KA | WT | WT_MPTP_EMPA | WT_EMPA | 0,000886 | 0,000116 | *** |
| KA | S3KO | S3KO_MPTP | S3KO | 0,273267 | 0,000133 |  |
| KA | S3KO | S3KO_EMPA | S3KO | 0,10515 | 0,000133 |  |
| KA | S3KO | S3KO_MPTP_EMPA | S3KO | 0,000583 | 0,000133 | *** |
| KA | S3KO | S3KO_MPTP | S3KO_MPTP | | 0,000133 |  |
| KA | S3KO | S3KO_EMPA | S3KO_MPTP | 0,889567 | 0,000133 |  |
| KA | S3KO | S3KO_MPTP_EMPA | S3KO_MPTP | 0,002485 | 0,000133 | ** |
| KA | S3KO | S3KO_MPTP | S3KO_EMPA | | 0,000133 |  |
| KA | S3KO | S3KO_EMPA | S3KO_EMPA | | 0,000133 |  |
| KA | S3KO | S3KO_MPTP_EMPA | S3KO_EMPA | 0,001402 | 0,000133 | ** |
| AA | WT | WT_MPTP | WT | 0,849338 | 0,001963 |  |
| AA | WT | WT_EMPA | WT | 0,051481 | 0,001963 |  |
| AA | WT | WT_MPTP_EMPA | WT | 0,004592 | 0,001963 | ** |
| AA | WT | WT_MPTP | WT_MPTP | | 0,001963 |  |
| AA | WT | WT_EMPA | WT_MPTP | 0,271891 | 0,001963 |  |
| AA | WT | WT_MPTP_EMPA | WT_MPTP | 0,011945 | 0,001963 | * |
| AA | WT | WT_MPTP | WT_EMPA | | 0,001963 |  |
| AA | WT | WT_EMPA | WT_EMPA | | 0,001963 |  |
| AA | WT | WT_MPTP_EMPA | WT_EMPA | 0,138558 | 0,001963 |  |
| AA | S3KO | S3KO_MPTP | S3KO | 0,9952 | 0,040934 |  |
| AA | S3KO | S3KO_EMPA | S3KO | 0,99865 | 0,040934 |  |
| AA | S3KO | S3KO_MPTP_EMPA | S3KO | 0,033667 | 0,040934 | * |
| AA | S3KO | S3KO_MPTP | S3KO_MPTP | | 0,040934 |  |
| AA | S3KO | S3KO_EMPA | S3KO_MPTP | 0,978692 | 0,040934 |  |
| AA | S3KO | S3KO_MPTP_EMPA | S3KO_MPTP | 0,036347 | 0,040934 | * |
| AA | S3KO | S3KO_MPTP | S3KO_EMPA | | 0,040934 |  |
| AA | S3KO | S3KO_EMPA | S3KO_EMPA | | 0,040934 |  |
| AA | S3KO | S3KO_MPTP_EMPA | S3KO_EMPA | 0,029931 | 0,040934 | * |
| 3OHK | WT | WT_MPTP | WT | 0,973801 | 0,208942 |  |
| 3OHK | WT | WT_EMPA | WT | 0,280124 | 0,208942 |  |
| 3OHK | WT | WT_MPTP_EMPA | WT | 0,945971 | 0,208942 |  |
| 3OHK | WT | WT_MPTP | WT_MPTP | | 0,208942 |  |
| 3OHK | WT | WT_EMPA | WT_MPTP | 0,616334 | 0,208942 |  |
| 3OHK | WT | WT_MPTP_EMPA | WT_MPTP | 0,835697 | 0,208942 |  |
| 3OHK | WT | WT_MPTP | WT_EMPA | | 0,208942 |  |
| 3OHK | WT | WT_EMPA | WT_EMPA | | 0,208942 |  |
| 3OHK | WT | WT_MPTP_EMPA | WT_EMPA | 0,282666 | 0,208942 |  |
| 3OHK | S3KO | S3KO_MPTP | S3KO | 0,42647 | 0,01488 |  |
| 3OHK | S3KO | S3KO_EMPA | S3KO | 0,032706 | 0,01488 | * |
| 3OHK | S3KO | S3KO_MPTP_EMPA | S3KO | 1 | 0,01488 |  |
| 3OHK | S3KO | S3KO_MPTP | S3KO_MPTP | | 0,01488 |  |
| 3OHK | S3KO | S3KO_EMPA | S3KO_MPTP | 0,097487 | 0,01488 |  |
| 3OHK | S3KO | S3KO_MPTP_EMPA | S3KO_MPTP | 0,525719 | 0,01488 |  |
| 3OHK | S3KO | S3KO_MPTP | S3KO_EMPA | | 0,01488 |  |
| 3OHK | S3KO | S3KO_EMPA | S3KO_EMPA | | 0,01488 |  |
| 3OHK | S3KO | S3KO_MPTP_EMPA | S3KO_EMPA | 0,046624 | 0,01488 | * |
| XA | WT | WT_MPTP | WT | 0,533659 | 0,102883 |  |
| XA | WT | WT_EMPA | WT | 0,992278 | 0,102883 |  |
| XA | WT | WT_MPTP_EMPA | WT | 0,122142 | 0,102883 |  |
| XA | WT | WT_MPTP | WT_MPTP | | 0,102883 |  |
| XA | WT | WT_EMPA | WT_MPTP | 0,668811 | 0,102883 |  |
| XA | WT | WT_MPTP_EMPA | WT_MPTP | 0,263669 | 0,102883 |  |
| XA | WT | WT_MPTP | WT_EMPA | | 0,102883 |  |
| XA | WT | WT_EMPA | WT_EMPA | | 0,102883 |  |
| XA | WT | WT_MPTP_EMPA | WT_EMPA | 0,139567 | 0,102883 |  |
| XA | S3KO | S3KO_MPTP | S3KO | 0,07887 | 0,001505 |  |
| XA | S3KO | S3KO_EMPA | S3KO | 0,161546 | 0,001505 |  |
| XA | S3KO | S3KO_MPTP_EMPA | S3KO | 0,010192 | 0,001505 | * |
| XA | S3KO | S3KO_MPTP | S3KO_MPTP | | 0,001505 |  |
| XA | S3KO | S3KO_EMPA | S3KO_MPTP | 0,530858 | 0,001505 |  |
| XA | S3KO | S3KO_MPTP_EMPA | S3KO_MPTP | 0,027589 | 0,001505 | * |
| XA | S3KO | S3KO_MPTP | S3KO_EMPA | | 0,001505 |  |
| XA | S3KO | S3KO_EMPA | S3KO_EMPA | | 0,001505 |  |
| XA | S3KO | S3KO_MPTP_EMPA | S3KO_EMPA | 0,246784 | 0,001505 |  |
| NAD | WT | WT_MPTP | WT | 0,964917 | 0,027044 |  |
| NAD | WT | WT_EMPA | WT | 0,044904 | 0,027044 | * |
| NAD | WT | WT_MPTP_EMPA | WT | 0,033478 | 0,027044 | * |
| NAD | WT | WT_MPTP | WT_MPTP | | 0,027044 |  |
| NAD | WT | WT_EMPA | WT_MPTP | 0,468122 | 0,027044 |  |
| NAD | WT | WT_MPTP_EMPA | WT_MPTP | 0,446937 | 0,027044 |  |
| NAD | WT | WT_MPTP | WT_EMPA | | 0,027044 |  |
| NAD | WT | WT_EMPA | WT_EMPA | | 0,027044 |  |
| NAD | WT | WT_MPTP_EMPA | WT_EMPA | 0,995541 | 0,027044 |  |
| NAD | S3KO | S3KO_MPTP | S3KO | 0,921576 | 0,107634 |  |
| NAD | S3KO | S3KO_EMPA | S3KO | 0,934286 | 0,107634 |  |
| NAD | S3KO | S3KO_MPTP_EMPA | S3KO | 0,546768 | 0,107634 |  |
| NAD | S3KO | S3KO_MPTP | S3KO_MPTP | | 0,107634 |  |
| NAD | S3KO | S3KO_EMPA | S3KO_MPTP | 0,531667 | 0,107634 |  |
| NAD | S3KO | S3KO_MPTP_EMPA | S3KO_MPTP | 0,155204 | 0,107634 |  |
| NAD | S3KO | S3KO_MPTP | S3KO_EMPA | | 0,107634 |  |
| NAD | S3KO | S3KO_EMPA | S3KO_EMPA | | 0,107634 |  |
| NAD | S3KO | S3KO_MPTP_EMPA | S3KO_EMPA | 0,568509 | 0,107634 |  |
| Oxidativ_Stress_Index | WT | WT_MPTP | WT | 0,479099 | 0,00965 |  |
| Oxidativ_Stress_Index | WT | WT_EMPA | WT | 0,610957 | 0,00965 |  |
| Oxidativ_Stress_Index | WT | WT_MPTP_EMPA | WT | 0,289589 | 0,00965 |  |
| Oxidativ_Stress_Index | WT | WT_MPTP | WT_MPTP | | 0,00965 |  |
| Oxidativ_Stress_Index | WT | WT_EMPA | WT_MPTP | 0,81779 | 0,00965 |  |
| Oxidativ_Stress_Index | WT | WT_MPTP_EMPA | WT_MPTP | 0,085583 | 0,00965 |  |
| Oxidativ_Stress_Index | WT | WT_MPTP | WT_EMPA | | 0,00965 |  |
| Oxidativ_Stress_Index | WT | WT_EMPA | WT_EMPA | | 0,00965 |  |
| Oxidativ_Stress_Index | WT | WT_MPTP_EMPA | WT_EMPA | 0,082901 | 0,00965 |  |
| Oxidativ_Stress_Index | S3KO | S3KO_MPTP | S3KO | 0,065046 | 0,001803 |  |
| Oxidativ_Stress_Index | S3KO | S3KO_EMPA | S3KO | 0,034066 | 0,001803 | * |
| Oxidativ_Stress_Index | S3KO | S3KO_MPTP_EMPA | S3KO | 0,025025 | 0,001803 | * |
| Oxidativ_Stress_Index | S3KO | S3KO_MPTP | S3KO_MPTP | | 0,001803 |  |
| Oxidativ_Stress_Index | S3KO | S3KO_EMPA | S3KO_MPTP | 0,369197 | 0,001803 |  |
| Oxidativ_Stress_Index | S3KO | S3KO_MPTP_EMPA | S3KO_MPTP | 0,022334 | 0,001803 | * |
| Oxidativ_Stress_Index | S3KO | S3KO_MPTP | S3KO_EMPA | | 0,001803 |  |
| Oxidativ_Stress_Index | S3KO | S3KO_EMPA | S3KO_EMPA | | 0,001803 |  |
| Oxidativ_Stress_Index | S3KO | S3KO_MPTP_EMPA | S3KO_EMPA | 0,33453 | 0,001803 |  |
| KYN/TRP_IDOs_TDO | WT | WT_MPTP | WT | 0,849598 | 0,750102 |  |
| KYN/TRP_IDOs_TDO | WT | WT_EMPA | WT | 0,953375 | 0,750102 |  |
| KYN/TRP_IDOs_TDO | WT | WT_MPTP_EMPA | WT | 0,978639 | 0,750102 |  |
| KYN/TRP_IDOs_TDO | WT | WT_MPTP | WT_MPTP | | 0,750102 |  |
| KYN/TRP_IDOs_TDO | WT | WT_EMPA | WT_MPTP | 0,995689 | 0,750102 |  |
| KYN/TRP_IDOs_TDO | WT | WT_MPTP_EMPA | WT_MPTP | 0,746295 | 0,750102 |  |
| KYN/TRP_IDOs_TDO | WT | WT_MPTP | WT_EMPA | | 0,750102 |  |
| KYN/TRP_IDOs_TDO | WT | WT_EMPA | WT_EMPA | | 0,750102 |  |
| KYN/TRP_IDOs_TDO | WT | WT_MPTP_EMPA | WT_EMPA | 0,863589 | 0,750102 |  |
| KYN/TRP_IDOs_TDO | S3KO | S3KO_MPTP | S3KO | 0,987647 | 0,990134 |  |
| KYN/TRP_IDOs_TDO | S3KO | S3KO_EMPA | S3KO | 0,990879 | 0,990134 |  |
| KYN/TRP_IDOs_TDO | S3KO | S3KO_MPTP_EMPA | S3KO | 0,99556 | 0,990134 |  |
| KYN/TRP_IDOs_TDO | S3KO | S3KO_MPTP | S3KO_MPTP | | 0,990134 |  |
| KYN/TRP_IDOs_TDO | S3KO | S3KO_EMPA | S3KO_MPTP | 0,999971 | 0,990134 |  |
| KYN/TRP_IDOs_TDO | S3KO | S3KO_MPTP_EMPA | S3KO_MPTP | 0,998342 | 0,990134 |  |
| KYN/TRP_IDOs_TDO | S3KO | S3KO_MPTP | S3KO_EMPA | | 0,990134 |  |
| KYN/TRP_IDOs_TDO | S3KO | S3KO_EMPA | S3KO_EMPA | | 0,990134 |  |
| KYN/TRP_IDOs_TDO | S3KO | S3KO_MPTP_EMPA | S3KO_EMPA | 0,999355 | 0,990134 |  |
| KA/TRP | WT | WT_MPTP | WT | 0,130798 | 0,005004 |  |
| KA/TRP | WT | WT_EMPA | WT | 0,854904 | 0,005004 |  |
| KA/TRP | WT | WT_MPTP_EMPA | WT | 0,01654 | 0,005004 | * |
| KA/TRP | WT | WT_MPTP | WT_MPTP | | 0,005004 |  |
| KA/TRP | WT | WT_EMPA | WT_MPTP | 0,912264 | 0,005004 |  |
| KA/TRP | WT | WT_MPTP_EMPA | WT_MPTP | 0,030728 | 0,005004 | * |
| KA/TRP | WT | WT_MPTP | WT_EMPA | | 0,005004 |  |
| KA/TRP | WT | WT_EMPA | WT_EMPA | | 0,005004 |  |
| KA/TRP | WT | WT_MPTP_EMPA | WT_EMPA | 0,022015 | 0,005004 | * |
| KA/TRP | S3KO | S3KO_MPTP | S3KO | 0,226924 | 0,000171 |  |
| KA/TRP | S3KO | S3KO_EMPA | S3KO | 0,221907 | 0,000171 |  |
| KA/TRP | S3KO | S3KO_MPTP_EMPA | S3KO | 0,000447 | 0,000171 | *** |
| KA/TRP | S3KO | S3KO_MPTP | S3KO_MPTP | | 0,000171 |  |
| KA/TRP | S3KO | S3KO_EMPA | S3KO_MPTP | 0,933452 | 0,000171 |  |
| KA/TRP | S3KO | S3KO_MPTP_EMPA | S3KO_MPTP | 0,030049 | 0,000171 | * |
| KA/TRP | S3KO | S3KO_MPTP | S3KO_EMPA | | 0,000171 |  |
| KA/TRP | S3KO | S3KO_EMPA | S3KO_EMPA | | 0,000171 |  |
| KA/TRP | S3KO | S3KO_MPTP_EMPA | S3KO_EMPA | 0,003213 | 0,000171 | ** |
| KA/KYN_KATs | WT | WT_MPTP | WT | 0,154083 | 0,005496 |  |
| KA/KYN_KATs | WT | WT_EMPA | WT | 0,909423 | 0,005496 |  |
| KA/KYN_KATs | WT | WT_MPTP_EMPA | WT | 0,014704 | 0,005496 | * |
| KA/KYN_KATs | WT | WT_MPTP | WT_MPTP | | 0,005496 |  |
| KA/KYN_KATs | WT | WT_EMPA | WT_MPTP | 0,902291 | 0,005496 |  |
| KA/KYN_KATs | WT | WT_MPTP_EMPA | WT_MPTP | 0,023504 | 0,005496 | * |
| KA/KYN_KATs | WT | WT_MPTP | WT_EMPA | | 0,005496 |  |
| KA/KYN_KATs | WT | WT_EMPA | WT_EMPA | | 0,005496 |  |
| KA/KYN_KATs | WT | WT_MPTP_EMPA | WT_EMPA | 0,018121 | 0,005496 | * |
| KA/KYN_KATs | S3KO | S3KO_MPTP | S3KO | 0,222857 | 0,003791 |  |
| KA/KYN_KATs | S3KO | S3KO_EMPA | S3KO | 0,114883 | 0,003791 |  |
| KA/KYN_KATs | S3KO | S3KO_MPTP_EMPA | S3KO | 0,013864 | 0,003791 | * |
| KA/KYN_KATs | S3KO | S3KO_MPTP | S3KO_MPTP | | 0,003791 |  |
| KA/KYN_KATs | S3KO | S3KO_EMPA | S3KO_MPTP | 0,92399 | 0,003791 |  |
| KA/KYN_KATs | S3KO | S3KO_MPTP_EMPA | S3KO_MPTP | 0,069622 | 0,003791 |  |
| KA/KYN_KATs | S3KO | S3KO_MPTP | S3KO_EMPA | | 0,003791 |  |
| KA/KYN_KATs | S3KO | S3KO_EMPA | S3KO_EMPA | | 0,003791 |  |
| KA/KYN_KATs | S3KO | S3KO_MPTP_EMPA | S3KO_EMPA | 0,03753 | 0,003791 | * |
| AA/KYN_KYNU | WT | WT_MPTP | WT | 0,756718 | 0,009893 |  |
| AA/KYN_KYNU | WT | WT_EMPA | WT | 0,168648 | 0,009893 |  |
| AA/KYN_KYNU | WT | WT_MPTP_EMPA | WT | 0,013217 | 0,009893 | * |
| AA/KYN_KYNU | WT | WT_MPTP | WT_MPTP | | 0,009893 |  |
| AA/KYN_KYNU | WT | WT_EMPA | WT_MPTP | 0,955957 | 0,009893 |  |
| AA/KYN_KYNU | WT | WT_MPTP_EMPA | WT_MPTP | 0,050827 | 0,009893 |  |
| AA/KYN_KYNU | WT | WT_MPTP | WT_EMPA | | 0,009893 |  |
| AA/KYN_KYNU | WT | WT_EMPA | WT_EMPA | | 0,009893 |  |
| AA/KYN_KYNU | WT | WT_MPTP_EMPA | WT_EMPA | 0,064108 | 0,009893 |  |
| AA/KYN_KYNU | S3KO | S3KO_MPTP | S3KO | 0,903644 | 0,145134 |  |
| AA/KYN_KYNU | S3KO | S3KO_EMPA | S3KO | 0,998425 | 0,145134 |  |
| AA/KYN_KYNU | S3KO | S3KO_MPTP_EMPA | S3KO | 0,139976 | 0,145134 |  |
| AA/KYN_KYNU | S3KO | S3KO_MPTP | S3KO_MPTP | | 0,145134 |  |
| AA/KYN_KYNU | S3KO | S3KO_EMPA | S3KO_MPTP | 0,822451 | 0,145134 |  |
| AA/KYN_KYNU | S3KO | S3KO_MPTP_EMPA | S3KO_MPTP | 0,254358 | 0,145134 |  |
| AA/KYN_KYNU | S3KO | S3KO_MPTP | S3KO_EMPA | | 0,145134 |  |
| AA/KYN_KYNU | S3KO | S3KO_EMPA | S3KO_EMPA | | 0,145134 |  |
| AA/KYN_KYNU | S3KO | S3KO_MPTP_EMPA | S3KO_EMPA | 0,115182 | 0,145134 |  |
| 3OHK/KYN_KMO | WT | WT_MPTP | WT | 0,994829 | 0,023827 |  |
| 3OHK/KYN_KMO | WT | WT_EMPA | WT | 0,017704 | 0,023827 | * |
| 3OHK/KYN_KMO | WT | WT_MPTP_EMPA | WT | 0,65087 | 0,023827 |  |
| 3OHK/KYN_KMO | WT | WT_MPTP | WT_MPTP | | 0,023827 |  |
| 3OHK/KYN_KMO | WT | WT_EMPA | WT_MPTP | 0,153164 | 0,023827 |  |
| 3OHK/KYN_KMO | WT | WT_MPTP_EMPA | WT_MPTP | 0,913433 | 0,023827 |  |
| 3OHK/KYN_KMO | WT | WT_MPTP | WT_EMPA | | 0,023827 |  |
| 3OHK/KYN_KMO | WT | WT_EMPA | WT_EMPA | | 0,023827 |  |
| 3OHK/KYN_KMO | WT | WT_MPTP_EMPA | WT_EMPA | 0,171326 | 0,023827 |  |
| 3OHK/KYN_KMO | S3KO | S3KO_MPTP | S3KO | 0,999975 | 0,087196 |  |
| 3OHK/KYN_KMO | S3KO | S3KO_EMPA | S3KO | 0,102862 | 0,087196 |  |
| 3OHK/KYN_KMO | S3KO | S3KO_MPTP_EMPA | S3KO | 0,773526 | 0,087196 |  |
| 3OHK/KYN_KMO | S3KO | S3KO_MPTP | S3KO_MPTP | | 0,087196 |  |
| 3OHK/KYN_KMO | S3KO | S3KO_EMPA | S3KO_MPTP | 0,178327 | 0,087196 |  |
| 3OHK/KYN_KMO | S3KO | S3KO_MPTP_EMPA | S3KO_MPTP | 0,853197 | 0,087196 |  |
| 3OHK/KYN_KMO | S3KO | S3KO_MPTP | S3KO_EMPA | | 0,087196 |  |
| 3OHK/KYN_KMO | S3KO | S3KO_EMPA | S3KO_EMPA | | 0,087196 |  |
| 3OHK/KYN_KMO | S3KO | S3KO_MPTP_EMPA | S3KO_EMPA | 0,509791 | 0,087196 |  |
| XA/3OHK_KATIII | WT | WT_MPTP | WT | 0,418498 | 0,074466 |  |
| XA/3OHK_KATIII | WT | WT_EMPA | WT | 0,99403 | 0,074466 |  |
| XA/3OHK_KATIII | WT | WT_MPTP_EMPA | WT | 0,093954 | 0,074466 |  |
| XA/3OHK_KATIII | WT | WT_MPTP | WT_MPTP | | 0,074466 |  |
| XA/3OHK_KATIII | WT | WT_EMPA | WT_MPTP | 0,531454 | 0,074466 |  |
| XA/3OHK_KATIII | WT | WT_MPTP_EMPA | WT_MPTP | 0,346906 | 0,074466 |  |
| XA/3OHK_KATIII | WT | WT_MPTP | WT_EMPA | | 0,074466 |  |
| XA/3OHK_KATIII | WT | WT_EMPA | WT_EMPA | | 0,074466 |  |
| XA/3OHK_KATIII | WT | WT_MPTP_EMPA | WT_EMPA | 0,111677 | 0,074466 |  |
| XA/3OHK_KATIII | S3KO | S3KO_MPTP | S3KO | 0,112146 | 0,003698 |  |
| XA/3OHK_KATIII | S3KO | S3KO_EMPA | S3KO | 0,140111 | 0,003698 |  |
| XA/3OHK_KATIII | S3KO | S3KO_MPTP_EMPA | S3KO | 0,02943 | 0,003698 | * |
| XA/3OHK_KATIII | S3KO | S3KO_MPTP | S3KO_MPTP | | 0,003698 |  |
| XA/3OHK_KATIII | S3KO | S3KO_EMPA | S3KO_MPTP | 0,366344 | 0,003698 |  |
| XA/3OHK_KATIII | S3KO | S3KO_MPTP_EMPA | S3KO_MPTP | 0,099032 | 0,003698 |  |
| XA/3OHK_KATIII | S3KO | S3KO_MPTP | S3KO_EMPA | | 0,003698 |  |
| XA/3OHK_KATIII | S3KO | S3KO_EMPA | S3KO_EMPA | | 0,003698 |  |
| XA/3OHK_KATIII | S3KO | S3KO_MPTP_EMPA | S3KO_EMPA | 0,970572 | 0,003698 |  |
| INS | WT | WT_MPTP | WT | 0,846887 | 0,009843 |  |
| INS | WT | WT_EMPA | WT | 0,173687 | 0,009843 |  |
| INS | WT | WT_MPTP_EMPA | WT | 0,061321 | 0,009843 |  |
| INS | WT | WT_MPTP | WT_MPTP | | 0,009843 |  |
| INS | WT | WT_EMPA | WT_MPTP | 0,092789 | 0,009843 |  |
| INS | WT | WT_MPTP_EMPA | WT_MPTP | 0,043915 | 0,009843 | * |
| INS | WT | WT_MPTP | WT_EMPA | | 0,009843 |  |
| INS | WT | WT_EMPA | WT_EMPA | | 0,009843 |  |
| INS | WT | WT_MPTP_EMPA | WT_EMPA | 0,480368 | 0,009843 |  |
| INS | S3KO | S3KO_MPTP | S3KO | 0,422266 | 0,067935 |  |
| INS | S3KO | S3KO_EMPA | S3KO | 0,600469 | 0,067935 |  |
| INS | S3KO | S3KO_MPTP_EMPA | S3KO | 0,749556 | 0,067935 |  |
| INS | S3KO | S3KO_MPTP | S3KO_MPTP | | 0,067935 |  |
| INS | S3KO | S3KO_EMPA | S3KO_MPTP | 0,63594 | 0,067935 |  |
| INS | S3KO | S3KO_MPTP_EMPA | S3KO_MPTP | 0,100789 | 0,067935 |  |
| INS | S3KO | S3KO_MPTP | S3KO_EMPA | | 0,067935 |  |
| INS | S3KO | S3KO_EMPA | S3KO_EMPA | | 0,067935 |  |
| INS | S3KO | S3KO_MPTP_EMPA | S3KO_EMPA | 0,156798 | 0,067935 |  |
| PLP | WT | WT_MPTP | WT | 0,64436 | 0,309194 |  |
| PLP | WT | WT_EMPA | WT | 0,980999 | 0,309194 |  |
| PLP | WT | WT_MPTP_EMPA | WT | 0,867709 | 0,309194 |  |
| PLP | WT | WT_MPTP | WT_MPTP | | 0,309194 |  |
| PLP | WT | WT_EMPA | WT_MPTP | 0,396247 | 0,309194 |  |
| PLP | WT | WT_MPTP_EMPA | WT_MPTP | 0,909773 | 0,309194 |  |
| PLP | WT | WT_MPTP | WT_EMPA | | 0,309194 |  |
| PLP | WT | WT_EMPA | WT_EMPA | | 0,309194 |  |
| PLP | WT | WT_MPTP_EMPA | WT_EMPA | 0,470114 | 0,309194 |  |
| PLP | S3KO | S3KO_MPTP | S3KO | 0,278468 | 0,018768 |  |
| PLP | S3KO | S3KO_EMPA | S3KO | 0,566449 | 0,018768 |  |
| PLP | S3KO | S3KO_MPTP_EMPA | S3KO | 0,834778 | 0,018768 |  |
| PLP | S3KO | S3KO_MPTP | S3KO_MPTP | | 0,018768 |  |
| PLP | S3KO | S3KO_EMPA | S3KO_MPTP | 0,014022 | 0,018768 | * |
| PLP | S3KO | S3KO_MPTP_EMPA | S3KO_MPTP | 0,031909 | 0,018768 | * |
| PLP | S3KO | S3KO_MPTP | S3KO_EMPA | | 0,018768 |  |
| PLP | S3KO | S3KO_EMPA | S3KO_EMPA | | 0,018768 |  |
| PLP | S3KO | S3KO_MPTP_EMPA | S3KO_EMPA | 0,828044 | 0,018768 |  |
| IAA | WT | WT_MPTP | WT | 0,003858 | 0,000183 | ** |
| IAA | WT | WT_EMPA | WT | 0,768605 | 0,000183 |  |
| IAA | WT | WT_MPTP_EMPA | WT | 0,654089 | 0,000183 |  |
| IAA | WT | WT_MPTP | WT_MPTP | | 0,000183 |  |
| IAA | WT | WT_EMPA | WT_MPTP | 0,005311 | 0,000183 | ** |
| IAA | WT | WT_MPTP_EMPA | WT_MPTP | 0,057639 | 0,000183 |  |
| IAA | WT | WT_MPTP | WT_EMPA | | 0,000183 |  |
| IAA | WT | WT_EMPA | WT_EMPA | | 0,000183 |  |
| IAA | WT | WT_MPTP_EMPA | WT_EMPA | 0,248932 | 0,000183 |  |
| IAA | S3KO | S3KO_MPTP | S3KO | 0,302784 | 0,185338 |  |
| IAA | S3KO | S3KO_EMPA | S3KO | 0,446419 | 0,185338 |  |
| IAA | S3KO | S3KO_MPTP_EMPA | S3KO | 0,308177 | 0,185338 |  |
| IAA | S3KO | S3KO_MPTP | S3KO_MPTP | | 0,185338 |  |
| IAA | S3KO | S3KO_EMPA | S3KO_MPTP | 0,503298 | 0,185338 |  |
| IAA | S3KO | S3KO_MPTP_EMPA | S3KO_MPTP | 0,999914 | 0,185338 |  |
| IAA | S3KO | S3KO_MPTP | S3KO_EMPA | | 0,185338 |  |
| IAA | S3KO | S3KO_EMPA | S3KO_EMPA | | 0,185338 |  |
| IAA | S3KO | S3KO_MPTP_EMPA | S3KO_EMPA | 0,51995 | 0,185338 |  |
| I3CA | WT | WT_MPTP | WT | 0,549441 | 0,589468 |  |
| I3CA | WT | WT_EMPA | WT | 0,998905 | 0,589468 |  |
| I3CA | WT | WT_MPTP_EMPA | WT | 0,893322 | 0,589468 |  |
| I3CA | WT | WT_MPTP | WT_MPTP | | 0,589468 |  |
| I3CA | WT | WT_EMPA | WT_MPTP | 0,66628 | 0,589468 |  |
| I3CA | WT | WT_MPTP_EMPA | WT_MPTP | 0,83199 | 0,589468 |  |
| I3CA | WT | WT_MPTP | WT_EMPA | | 0,589468 |  |
| I3CA | WT | WT_EMPA | WT_EMPA | | 0,589468 |  |
| I3CA | WT | WT_MPTP_EMPA | WT_EMPA | 0,956797 | 0,589468 |  |
| I3CA | S3KO | S3KO_MPTP | S3KO | 0,92145 | 0,296407 |  |
| I3CA | S3KO | S3KO_EMPA | S3KO | 0,374992 | 0,296407 |  |
| I3CA | S3KO | S3KO_MPTP_EMPA | S3KO | 0,442798 | 0,296407 |  |
| I3CA | S3KO | S3KO_MPTP | S3KO_MPTP | | 0,296407 |  |
| I3CA | S3KO | S3KO_EMPA | S3KO_MPTP | 0,483571 | 0,296407 |  |
| I3CA | S3KO | S3KO_MPTP_EMPA | S3KO_MPTP | 0,590657 | 0,296407 |  |
| I3CA | S3KO | S3KO_MPTP | S3KO_EMPA | | 0,296407 |  |
| I3CA | S3KO | S3KO_EMPA | S3KO_EMPA | | 0,296407 |  |
| I3CA | S3KO | S3KO_MPTP_EMPA | S3KO_EMPA | 0,999528 | 0,296407 |  |
| ILA | WT | WT_MPTP | WT | 0,505535 | 0,043032 |  |
| ILA | WT | WT_EMPA | WT | 0,455659 | 0,043032 |  |
| ILA | WT | WT_MPTP_EMPA | WT | 0,984711 | 0,043032 |  |
| ILA | WT | WT_MPTP | WT_MPTP | | 0,043032 |  |
| ILA | WT | WT_EMPA | WT_MPTP | 0,079715 | 0,043032 |  |
| ILA | WT | WT_MPTP_EMPA | WT_MPTP | 0,319997 | 0,043032 |  |
| ILA | WT | WT_MPTP | WT_EMPA | | 0,043032 |  |
| ILA | WT | WT_EMPA | WT_EMPA | | 0,043032 |  |
| ILA | WT | WT_MPTP_EMPA | WT_EMPA | 0,639367 | 0,043032 |  |
| ILA | S3KO | S3KO_MPTP | S3KO | 0,999977 | 0,573623 |  |
| ILA | S3KO | S3KO_EMPA | S3KO | 0,972944 | 0,573623 |  |
| ILA | S3KO | S3KO_MPTP_EMPA | S3KO | 0,553399 | 0,573623 |  |
| ILA | S3KO | S3KO_MPTP | S3KO_MPTP | | 0,573623 |  |
| ILA | S3KO | S3KO_EMPA | S3KO_MPTP | 0,97983 | 0,573623 |  |
| ILA | S3KO | S3KO_MPTP_EMPA | S3KO_MPTP | 0,609233 | 0,573623 |  |
| ILA | S3KO | S3KO_MPTP | S3KO_EMPA | | 0,573623 |  |
| ILA | S3KO | S3KO_EMPA | S3KO_EMPA | | 0,573623 |  |
| ILA | S3KO | S3KO_MPTP_EMPA | S3KO_EMPA | 0,46954 | 0,573623 |  |
| IPA | WT | WT_MPTP | WT | 0,995136 | 0,639108 |  |
| IPA | WT | WT_EMPA | WT | 0,861464 | 0,639108 |  |
| IPA | WT | WT_MPTP_EMPA | WT | 0,967215 | 0,639108 |  |
| IPA | WT | WT_MPTP | WT_MPTP | | 0,639108 |  |
| IPA | WT | WT_EMPA | WT_MPTP | 0,907666 | 0,639108 |  |
| IPA | WT | WT_MPTP_EMPA | WT_MPTP | 0,883093 | 0,639108 |  |
| IPA | WT | WT_MPTP | WT_EMPA | | 0,639108 |  |
| IPA | WT | WT_EMPA | WT_EMPA | | 0,639108 |  |
| IPA | WT | WT_MPTP_EMPA | WT_EMPA | 0,611681 | 0,639108 |  |
| IPA | S3KO | S3KO_MPTP | S3KO | 0,398802 | 0,436275 |  |
| IPA | S3KO | S3KO_EMPA | S3KO | 0,482513 | 0,436275 |  |
| IPA | S3KO | S3KO_MPTP_EMPA | S3KO | 0,696064 | 0,436275 |  |
| IPA | S3KO | S3KO_MPTP | S3KO_MPTP | | 0,436275 |  |
| IPA | S3KO | S3KO_EMPA | S3KO_MPTP | 0,965476 | 0,436275 |  |
| IPA | S3KO | S3KO_MPTP_EMPA | S3KO_MPTP | 0,848612 | 0,436275 |  |
| IPA | S3KO | S3KO_MPTP | S3KO_EMPA | | 0,436275 |  |
| IPA | S3KO | S3KO_EMPA | S3KO_EMPA | | 0,436275 |  |
| IPA | S3KO | S3KO_MPTP_EMPA | S3KO_EMPA | 0,956033 | 0,436275 |  |
| pCS | WT | WT_MPTP | WT | 0,110536 | 0,012765 |  |
| pCS | WT | WT_EMPA | WT | 0,260961 | 0,012765 |  |
| pCS | WT | WT_MPTP_EMPA | WT | 0,999212 | 0,012765 |  |
| pCS | WT | WT_MPTP | WT_MPTP | | 0,012765 |  |
| pCS | WT | WT_EMPA | WT_MPTP | 0,690512 | 0,012765 |  |
| pCS | WT | WT_MPTP_EMPA | WT_MPTP | 0,048306 | 0,012765 | * |
| pCS | WT | WT_MPTP | WT_EMPA | | 0,012765 |  |
| pCS | WT | WT_EMPA | WT_EMPA | | 0,012765 |  |
| pCS | WT | WT_MPTP_EMPA | WT_EMPA | 0,132655 | 0,012765 |  |
| pCS | S3KO | S3KO_MPTP | S3KO | 0,813915 | 0,109879 |  |
| pCS | S3KO | S3KO_EMPA | S3KO | 0,937573 | 0,109879 |  |
| pCS | S3KO | S3KO_MPTP_EMPA | S3KO | 0,995599 | 0,109879 |  |
| pCS | S3KO | S3KO_MPTP | S3KO_MPTP | | 0,109879 |  |
| pCS | S3KO | S3KO_EMPA | S3KO_MPTP | 0,767507 | 0,109879 |  |
| pCS | S3KO | S3KO_MPTP_EMPA | S3KO_MPTP | 0,096404 | 0,109879 |  |
| pCS | S3KO | S3KO_MPTP | S3KO_EMPA | | 0,109879 |  |
| pCS | S3KO | S3KO_EMPA | S3KO_EMPA | | 0,109879 |  |
| pCS | S3KO | S3KO_MPTP_EMPA | S3KO_EMPA | 0,363854 | 0,109879 |  |
| INS/TRP_TNA | WT | WT_MPTP | WT | 0,962223 | 0,073051 |  |
| INS/TRP_TNA | WT | WT_EMPA | WT | 0,364961 | 0,073051 |  |
| INS/TRP_TNA | WT | WT_MPTP_EMPA | WT | 0,239441 | 0,073051 |  |
| INS/TRP_TNA | WT | WT_MPTP | WT_MPTP | | 0,073051 |  |
| INS/TRP_TNA | WT | WT_EMPA | WT_MPTP | 0,193181 | 0,073051 |  |
| INS/TRP_TNA | WT | WT_MPTP_EMPA | WT_MPTP | 0,205944 | 0,073051 |  |
| INS/TRP_TNA | WT | WT_MPTP | WT_EMPA | | 0,073051 |  |
| INS/TRP_TNA | WT | WT_EMPA | WT_EMPA | | 0,073051 |  |
| INS/TRP_TNA | WT | WT_MPTP_EMPA | WT_EMPA | 0,50022 | 0,073051 |  |
| INS/TRP_TNA | S3KO | S3KO_MPTP | S3KO | 0,493888 | 0,067628 |  |
| INS/TRP_TNA | S3KO | S3KO_EMPA | S3KO | 0,73865 | 0,067628 |  |
| INS/TRP_TNA | S3KO | S3KO_MPTP_EMPA | S3KO | 0,973575 | 0,067628 |  |
| INS/TRP_TNA | S3KO | S3KO_MPTP | S3KO_MPTP | | 0,067628 |  |
| INS/TRP_TNA | S3KO | S3KO_EMPA | S3KO_MPTP | 0,683462 | 0,067628 |  |
| INS/TRP_TNA | S3KO | S3KO_MPTP_EMPA | S3KO_MPTP | 0,072671 | 0,067628 |  |
| INS/TRP_TNA | S3KO | S3KO_MPTP | S3KO_EMPA | | 0,067628 |  |
| INS/TRP_TNA | S3KO | S3KO_EMPA | S3KO_EMPA | | 0,067628 |  |
| INS/TRP_TNA | S3KO | S3KO_MPTP_EMPA | S3KO_EMPA | 0,220038 | 0,067628 |  |
| IAA/TRP_TMO_TrD_ArAT | WT | WT_MPTP | WT | 0,006069 | 0,000904 | ** |
| IAA/TRP_TMO_TrD_ArAT | WT | WT_EMPA | WT | 0,940331 | 0,000904 |  |
| IAA/TRP_TMO_TrD_ArAT | WT | WT_MPTP_EMPA | WT | 0,131359 | 0,000904 |  |
| IAA/TRP_TMO_TrD_ArAT | WT | WT_MPTP | WT_MPTP | | 0,000904 |  |
| IAA/TRP_TMO_TrD_ArAT | WT | WT_EMPA | WT_MPTP | 0,004387 | 0,000904 | ** |
| IAA/TRP_TMO_TrD_ArAT | WT | WT_MPTP_EMPA | WT_MPTP | 0,26542 | 0,000904 |  |
| IAA/TRP_TMO_TrD_ArAT | WT | WT_MPTP | WT_EMPA | | 0,000904 |  |
| IAA/TRP_TMO_TrD_ArAT | WT | WT_EMPA | WT_EMPA | | 0,000904 |  |
| IAA/TRP_TMO_TrD_ArAT | WT | WT_MPTP_EMPA | WT_EMPA | 0,207851 | 0,000904 |  |
| IAA/TRP_TMO_TrD_ArAT | S3KO | S3KO_MPTP | S3KO | 0,146453 | 0,0243 |  |
| IAA/TRP_TMO_TrD_ArAT | S3KO | S3KO_EMPA | S3KO | 0,410438 | 0,0243 |  |
| IAA/TRP_TMO_TrD_ArAT | S3KO | S3KO_MPTP_EMPA | S3KO | 0,077498 | 0,0243 |  |
| IAA/TRP_TMO_TrD_ArAT | S3KO | S3KO_MPTP | S3KO_MPTP | | 0,0243 |  |
| IAA/TRP_TMO_TrD_ArAT | S3KO | S3KO_EMPA | S3KO_MPTP | 0,497088 | 0,0243 |  |
| IAA/TRP_TMO_TrD_ArAT | S3KO | S3KO_MPTP_EMPA | S3KO_MPTP | 0,509167 | 0,0243 |  |
| IAA/TRP_TMO_TrD_ArAT | S3KO | S3KO_MPTP | S3KO_EMPA | | 0,0243 |  |
| IAA/TRP_TMO_TrD_ArAT | S3KO | S3KO_EMPA | S3KO_EMPA | | 0,0243 |  |
| IAA/TRP_TMO_TrD_ArAT | S3KO | S3KO_MPTP_EMPA | S3KO_EMPA | 0,113764 | 0,0243 |  |
| pCS/TYR | WT | WT_MPTP | WT | 0,071927 | 0,022323 |  |
| pCS/TYR | WT | WT_EMPA | WT | 0,058591 | 0,022323 |  |
| pCS/TYR | WT | WT_MPTP_EMPA | WT | 0,997904 | 0,022323 |  |
| pCS/TYR | WT | WT_MPTP | WT_MPTP | | 0,022323 |  |
| pCS/TYR | WT | WT_EMPA | WT_MPTP | 0,966834 | 0,022323 |  |
| pCS/TYR | WT | WT_MPTP_EMPA | WT_MPTP | 0,170976 | 0,022323 |  |
| pCS/TYR | WT | WT_MPTP | WT_EMPA | | 0,022323 |  |
| pCS/TYR | WT | WT_EMPA | WT_EMPA | | 0,022323 |  |
| pCS/TYR | WT | WT_MPTP_EMPA | WT_EMPA | 0,145033 | 0,022323 |  |
| pCS/TYR | S3KO | S3KO_MPTP | S3KO | 0,743492 | 0,347496 |  |
| pCS/TYR | S3KO | S3KO_EMPA | S3KO | 0,871833 | 0,347496 |  |
| pCS/TYR | S3KO | S3KO_MPTP_EMPA | S3KO | 0,977943 | 0,347496 |  |
| pCS/TYR | S3KO | S3KO_MPTP | S3KO_MPTP | | 0,347496 |  |
| pCS/TYR | S3KO | S3KO_EMPA | S3KO_MPTP | 0,750416 | 0,347496 |  |
| pCS/TYR | S3KO | S3KO_MPTP_EMPA | S3KO_MPTP | 0,469406 | 0,347496 |  |
| pCS/TYR | S3KO | S3KO_MPTP | S3KO_EMPA | | 0,347496 |  |
| pCS/TYR | S3KO | S3KO_EMPA | S3KO_EMPA | | 0,347496 |  |
| pCS/TYR | S3KO | S3KO_MPTP_EMPA | S3KO_EMPA | 0,608029 | 0,347496 |  |
| GSH | WT | WT_MPTP | WT | 0,87581 | 0,341793 |  |
| GSH | WT | WT_EMPA | WT | 0,405205 | 0,341793 |  |
| GSH | WT | WT_MPTP_EMPA | WT | 0,349957 | 0,341793 |  |
| GSH | WT | WT_MPTP | WT_MPTP | | 0,341793 |  |
| GSH | WT | WT_EMPA | WT_MPTP | 0,87524 | 0,341793 |  |
| GSH | WT | WT_MPTP_EMPA | WT_MPTP | 0,741625 | 0,341793 |  |
| GSH | WT | WT_MPTP | WT_EMPA | | 0,341793 |  |
| GSH | WT | WT_EMPA | WT_EMPA | | 0,341793 |  |
| GSH | WT | WT_MPTP_EMPA | WT_EMPA | 0,970272 | 0,341793 |  |
| GSH | S3KO | S3KO_MPTP | S3KO | 0,608272 | 0,037926 |  |
| GSH | S3KO | S3KO_EMPA | S3KO | 0,999879 | 0,037926 |  |
| GSH | S3KO | S3KO_MPTP_EMPA | S3KO | 0,193427 | 0,037926 |  |
| GSH | S3KO | S3KO_MPTP | S3KO_MPTP | | 0,037926 |  |
| GSH | S3KO | S3KO_EMPA | S3KO_MPTP | 0,504935 | 0,037926 |  |
| GSH | S3KO | S3KO_MPTP_EMPA | S3KO_MPTP | 0,014432 | 0,037926 | * |
| GSH | S3KO | S3KO_MPTP | S3KO_EMPA | | 0,037926 |  |
| GSH | S3KO | S3KO_EMPA | S3KO_EMPA | | 0,037926 |  |
| GSH | S3KO | S3KO_MPTP_EMPA | S3KO_EMPA | 0,158962 | 0,037926 |  |
| GSSG | WT | WT_MPTP | WT | 0,336233 | 0,384706 |  |
| GSSG | WT | WT_EMPA | WT | 0,47009 | 0,384706 |  |
| GSSG | WT | WT_MPTP_EMPA | WT | 0,385378 | 0,384706 |  |
| GSSG | WT | WT_MPTP | WT_MPTP | | 0,384706 |  |
| GSSG | WT | WT_EMPA | WT_MPTP | 0,99972 | 0,384706 |  |
| GSSG | WT | WT_MPTP_EMPA | WT_MPTP | 0,999996 | 0,384706 |  |
| GSSG | WT | WT_MPTP | WT_EMPA | | 0,384706 |  |
| GSSG | WT | WT_EMPA | WT_EMPA | | 0,384706 |  |
| GSSG | WT | WT_MPTP_EMPA | WT_EMPA | 0,999615 | 0,384706 |  |
| GSSG | S3KO | S3KO_MPTP | S3KO | 0,99955 | 0,001091 |  |
| GSSG | S3KO | S3KO_EMPA | S3KO | 0,758902 | 0,001091 |  |
| GSSG | S3KO | S3KO_MPTP_EMPA | S3KO | 0,388404 | 0,001091 |  |
| GSSG | S3KO | S3KO_MPTP | S3KO_MPTP | | 0,001091 |  |
| GSSG | S3KO | S3KO_EMPA | S3KO_MPTP | 0,117328 | 0,001091 |  |
| GSSG | S3KO | S3KO_MPTP_EMPA | S3KO_MPTP | 0,000642 | 0,001091 | *** |
| GSSG | S3KO | S3KO_MPTP | S3KO_EMPA | | 0,001091 |  |
| GSSG | S3KO | S3KO_EMPA | S3KO_EMPA | | 0,001091 |  |
| GSSG | S3KO | S3KO_MPTP_EMPA | S3KO_EMPA | 0,112881 | 0,001091 |  |
| GSH/GSSG | WT | WT_MPTP | WT | 0,766969 | 0,263298 |  |
| GSH/GSSG | WT | WT_EMPA | WT | 0,345795 | 0,263298 |  |
| GSH/GSSG | WT | WT_MPTP_EMPA | WT | 0,626988 | 0,263298 |  |
| GSH/GSSG | WT | WT_MPTP | WT_MPTP | | 0,263298 |  |
| GSH/GSSG | WT | WT_EMPA | WT_MPTP | 0,508067 | 0,263298 |  |
| GSH/GSSG | WT | WT_MPTP_EMPA | WT_MPTP | 0,757357 | 0,263298 |  |
| GSH/GSSG | WT | WT_MPTP | WT_EMPA | | 0,263298 |  |
| GSH/GSSG | WT | WT_EMPA | WT_EMPA | | 0,263298 |  |
| GSH/GSSG | WT | WT_MPTP_EMPA | WT_EMPA | 1 | 0,263298 |  |
| GSH/GSSG | S3KO | S3KO_MPTP | S3KO | 0,866506 | 0,022803 |  |
| GSH/GSSG | S3KO | S3KO_EMPA | S3KO | 0,999999 | 0,022803 |  |
| GSH/GSSG | S3KO | S3KO_MPTP_EMPA | S3KO | 0,815476 | 0,022803 |  |
| GSH/GSSG | S3KO | S3KO_MPTP | S3KO_MPTP | | 0,022803 |  |
| GSH/GSSG | S3KO | S3KO_EMPA | S3KO_MPTP | 0,524423 | 0,022803 |  |
| GSH/GSSG | S3KO | S3KO_MPTP_EMPA | S3KO_MPTP | 0,005254 | 0,022803 | ** |
| GSH/GSSG | S3KO | S3KO_MPTP | S3KO_EMPA | | 0,022803 |  |
| GSH/GSSG | S3KO | S3KO_EMPA | S3KO_EMPA | | 0,022803 |  |
| GSH/GSSG | S3KO | S3KO_MPTP_EMPA | S3KO_EMPA | 0,432383 | 0,022803 |  |
